# High Serum Vitamin D Concentrations, Induced *via* Diet, Trigger Immune and Intestinal Microbiota Alterations Leading to Type 1 Diabetes Protection in NOD Mice

**DOI:** 10.3389/fimmu.2022.902678

**Published:** 2022-06-09

**Authors:** Pieter-Jan Martens, Javier Centelles-Lodeiro, Darcy Ellis, Dana Paulina Cook, Gabriele Sassi, Lieve Verlinden, Annemieke Verstuyf, Jeroen Raes, Chantal Mathieu, Conny Gysemans

**Affiliations:** ^1^ Clinical and Experimental Endocrinology (CEE), Katholieke Universiteit Leuven, Leuven, Belgium; ^2^ Laboratory of Molecular Bacteriology, Rega-Institute, Katholieke Universiteit (KU) Leuven, Leuven, Belgium

**Keywords:** type 1 diabetes, vitamin D, immunomodulation, gut microbiota, gut permeability, microbiome

## Abstract

The hormonally-active form of vitamin D, 1,25-dihydroxyvitamin D_3_, can modulate both innate and adaptive immunity, through binding to the nuclear vitamin D receptor expressed in most immune cells. A high dose of regular vitamin D protected non-obese diabetic (NOD) mice against type 1 diabetes (T1D), when initiated at birth and given lifelong. However, considerable controversy exists on the level of circulating vitamin D (25-hydroxyvitamin D_3_, 25(OH)D_3_) needed to modulate the immune system in autoimmune-prone subjects and protect against T1D onset. Here, we evaluated the impact of two doses of dietary vitamin D supplementation (400 and 800 IU/day), given to female NOD mice from 3 until 25 weeks of age, on disease development, peripheral and gut immune system, gut epithelial barrier function, and gut bacterial taxonomy. Whereas serum 25(OH)D_3_ concentrations were 2.6- (400 IU/day) and 3.9-fold (800 IU/day) higher with dietary vitamin D supplementation compared to normal chow (NC), only the 800 IU/day vitamin D-supplemented diet delayed and reduced T1D incidence compared to NC. Flow cytometry analyses revealed an increased frequency of FoxP3^+^ Treg cells in the spleen of mice receiving the 800 IU/day vitamin D-supplemented diet. This vitamin D-induced increase in FoxP3^+^ Treg cells, also expressing the ecto-5’-nucleotidase CD73, only persisted in the spleen of mice at 25 weeks of age. At this time point, the frequency of IL-10-secreting CD4^+^ T cells was also increased in all studied immune organs. High-dose vitamin D supplementation was unable to correct gut leakiness nor did it significantly modify the increased gut microbial diversity and richness over time observed in NOD mice receiving NC. Intriguingly, the rise in alpha-diversity during maturation occurred especially in mice not progressing to hyperglycaemia. Principal coordinates analysis identified that both diet and disease status significantly influenced the inter-individual microbiota variation at the genus level. The abundance of the genera *Ruminoclostridium_9* and *Marvinbryantia* gradually increased or decreased, respectively in faecal samples of mice on the 800 IU/day vitamin D-supplemented diet compared to mice on the 400 IU/day vitamin D-supplemented diet or NC, irrespective of disease outcome. In summary, dietary vitamin D reduced T1D incidence in female NOD mice at a dose of 800, but not of 400, IU/day, and was accompanied by an expansion of Treg cells in various lymphoid organs and an altered intestinal microbiota signature.

## Introduction

The increase in the incidence of type 1 diabetes (T1D), the most common chronic immune-mediated disease in young children and adolescents, over the last 40 years cannot be explained by genetic drift but is probably related to one or more environmental exposures. The ‘hygiene hypothesis’ proposes that fewer early childhood infections (1), and poorer gut microbial diversity may deviate the immune system towards islet auto-reactivity (2, 3). Interestingly, enteroviruses (e.g., coxsackievirus B4 and rotavirus) have tropism for self-pancreatic tissues (4), and can directly instigate failure and complete destruction of the insulin-producing β cells (5). Moreover, T1D patients have enterovirus persistence associated with strong inflammation in their gut mucosa, which may promote islet autoimmunity by bystander activation mechanisms (6). The intestinal microflora in people with T1D seems less diverse, at both the abundance and functional level, compared to healthy individuals but whether this is cause or consequence is still under debate [reviewed in (7)]. Exactly how commensal bacteria and the immune system interact to provoke islet autoimmunity and ensuing T1D remains largely unknown. Intestinal epithelial barrier dysfunction may allow dietary antigens but also foreign microbial components to translocate into the periphery where they could subsequently trigger islet autoimmunity due to epitope mimicry (8–10).

Researchers also postulate that a lack of sun exposure and consequential vitamin D deficiency could lead to aberrant immune responses and more autoimmunity. Vitamin D deficiency is common among children with T1D and children with multiple islet autoantibodies compared to autoantibody-negative children, implying that it proceeds disease onset (11). In addition, we demonstrated that severe vitamin D deficiency in diabetes-prone non-obese diabetic (NOD) mice aggravated T1D development and was associated with immune system defects (12). Vitamin D is not only vital for calcium homeostasis and bone growth, but strong evidence points to its essential role in the functionality of both innate and adaptive immunity (13, 14). Its hormonally-active form, 1,25-dihydroxyvitamin D_3_ (1,25(OH)_2_D_3_), has powerful anti-inflammatory and immunomodulatory properties and functions by binding to the nuclear vitamin D receptor (VDR), present in most immune cell types [reviewed in (14, 15)]. Within the immune system, vitamin D has the ability to tolerise dendritic cells, inhibit IFN-γ- and IL-17-producing effector T (Teff) cells, induce regulatory CD4^+^ T (Treg) cells, and stimulate production of antimicrobial peptides [reviewed in (13, 14)]. Early-life treatment with 1,25(OH)_2_D_3_ protected against T1D development in NOD mice, although this regimen was associated with severe hypercalcemia and bone fractures (16). On the other hand, high-dose regular (dietary) vitamin D supplementation safely prevented T1D progression in NOD mice, but only when administered early and lifelong (17). Protection was associated with skewing of the Teff-to-Treg balance in favour of Treg cells in the pancreatic draining lymph nodes. Moreover, in humans, early life supplementation with regular vitamin D at a dose of 2,000 IU/day (50 µg/day) reduced the risk of developing T1D, up to an 80% decrease projected over the following 30 years (18). Based on these preclinical and observational data, a clinical trial was designed to study whether new-borns with an increased genetic risk for T1D will be protected from disease development when given 2,000 IU/day of regular vitamin D instead of the recommended 400 IU/day dose (ClinicalTrials.gov identifier: NCT00141986) (19). There remains however, a lot of discussion about the correct vitamin D substitution regimen and the (circulating) vitamin D concentrations needed to maintain immune homeostasis, as current practical guidelines are primarily focussing on bone health (20).

In regards to intestinal features, both vitamin D and its receptor have been reported to influence the gut commensal composition, maintain gut epithelial barrier function, and avoid pathogenic immune responses in the gut by inhibiting Teff responses and inducing FoxP3^+^ Treg cells [reviewed in (21)]. Vitamin D deficiency has been associated with a dysbiosed gut microbiota (22), altered gut mucosal defence (23), impaired mucus, and increased gut permeability (23, 24). Moreover, vitamin D supplementation in vitamin D-deficient women significantly increased gut microbial diversity and richness with an increase in the *Bacteroidetes* to *Firmicutes* ratio, along with an increase in the abundance of particular health-promoting taxa (22). Interestingly, repeated UVB light exposure increased alpha- and beta-diversity in the gut microbiota of subjects that had not taken vitamin D supplements prior to study enrolment (25). At the genus level, vitamin D seems to connect to some genera of the *Lachnospiraceae* family (25, 26). Circulating 25-hydroxyvitamin D_3_ (25(OH)D_3_) concentrations showed a strong correlation with the relative abundance of *Lachnospiraceae* genera (25). A recent genome-wide association study (GWAS) study established significant associations between gut microbial features and the VDR gene (27). Interestingly, the expression level of the VDR gene is not only regulated by vitamin D but also by other hormonal components, like secondary bile acids and metabolites produced by the gut microbiota (28, 29).

The complex interplay between vitamin D, the immune system, and the gut microbiota is clearly understudied and not much is known about how vitamin D supplementation affects the intestinal characteristics in autoimmune-prone subjects. In the current study, we evaluated the impact of two different doses of vitamin D supplementation (400 and 800 IU/day), administered lifelong *via* the diet, on T1D development and studied whether vitamin D intake and circulating concentrations of 25(OH)D_3_ were associated with peripheral and gut immunity (i.e., FoxP3^+^ Treg cells, CD39^+^CD73^+^ T cells, IL-10-secreting T cells, and T regulatory type 1 (Tr1) cells), intestinal barrier function, and gut microbiota composition in diabetes-prone NOD mice. We found that only the 800 IU/day vitamin D-supplemented diet, which increased 25(OH)D_3_ serum concentration up to a mean value of 193.9 nmol/L, delayed disease onset, significantly reduced T1D incidence, and was accompanied by an increased frequency of Treg cells in the studied immune organs, and an altered intestinal microbiota composition favouring *Ruminiclostridium_9* and diminishing *Marvinbryantia* at the genus level.

## Materials and Methods

### Animals and Experimental Design

The NOD Leuven strain is bred in specific pathogen free (SPF) environment and maintained under semi-barrier conditions in the animal facility of the KU Leuven since 1989. For this study, 93 female NOD mice were randomly assigned at the time of weaning to a grid cage and group housed (5/cage) per dietary condition to either a vitamin D-sufficient diet (normal chow, NC) or two different doses (400 or 800 IU/day) of a vitamin D-supplemented diet until 25 weeks of age. In NC the natural-ingredient was ssniff^®^ R/M-H maintenance diet containing 1% calcium, 0.7% phosphorus, and 1,000 IU vitamin D/kg diet (BioServices BV, Uden, the Netherlands), meeting the recommended concentrations for rodents according to the criteria described in the ‘Nutrient Requirements of Laboratory Animals’ (30). Based on a daily consumption of 4 g food per 20 g body weight, this corresponds to an intake of 4 IU/day of vitamin D. The 400 and 800 IU/day vitamin D-supplemented diet was the ssniff^®^ R/M-H maintenance diet containing 1% calcium, 0.7% phosphorus but supplemented with 100,000 or 200,000 IU vitamin D/kg diet, respectively. The animals had *ad libitum* access to both food and water. Mice were screened three times weekly for diabetes onset by evaluating glucose concentrations in urine (Diastix; Ascensia Diabetes Care, Machelen, Belgium) and venous blood (Accu-Chek; Roche Diagnostics, Vilvoorde, Belgium). Mice were diagnosed as diabetic (progressor mice; P) when having positive glycosuria and two consecutive blood glucose measurements above 200 mg/dL. The remaining mice that maintained normal blood glucose concentrations were labelled as non-progressor (NP) mice (**Supplementary Figure 1**).

### Serum Vitamin D, Calcium, and Phosphate Measurements

At 8 weeks of age, blood was collected by submandibular vein puncture. Serum or plasma was stored at –80°C until biochemical determinations were performed. Serum 25(OH)D_3_, 1,25(OH)_2_D_3_ and both serum and plasma 24,25(OH)_2_D_3_ levels were analysed using a liquid chromatography tandem mass spectrometry (LC-MS/MS) method (Sciex, Framingham, MA) (31). Serum calcium (product code: OSR60117) and phosphate (product code: OSR6122) were analysed on a Beckman Coulter DxC 700 AU chemistry analyser (Analis, Vilvoorde, Belgium).

### Flow Cytometric Analysis

Single-cell suspensions of spleen, pancreatic lymph nodes (PLN), and mesenteric lymph nodes (MLN) were prepared by mechanical disruption from mice at 8 and 25 weeks of age. The following antibodies were used: CD4, CD25, CD39, CD49b, CD73, lymphocyte-activation gene 3 (LAG3), and IL-10 (eBiosciences, Fisher Scientific SPRL, Merelbeke, Belgium). Intracellular staining was performed with FoxP3/Transcription Factor Staining Buffer Set (eBioscience). Cells were acquired on a Canto II AIG flow cytometer (BD Biosciences, Erembodegem, Belgium) and analysed with FlowJo software (FlowJo, LLC, Ashland, OR). All analyses were performed on fixable viability dye negative singlet population as outlined in the gating strategy (**Supplementary Figure 2**).

### Intestinal Barrier Function Using FITC-Dextran Assay

Dextran sulfate sodium (molecular mass of 70,000 Da, DSS, Fisher Scientific SPRL) was added at 5% (w/v) in drinking water *ad libitum* for 8 days to 8-week-old C57BL/6 mice, which has been described to destroy the intestinal barrier function and result in intestinal permeability and colitis-like symptoms. All mice including the DSS-treated C57BL/6 mice (as positive control) were food and water-starved overnight and kept in a cage without bedding to limit the coprophagic behaviour. FITC-dextran (Sigma-Aldrich, Overijse, Belgium) dissolved in PBS (100 mg/mL) was administered to each mouse (44 mg/100 g body weight) by oral gavage. After 4 hours, mice were sacrificed, serum was collected and FITC-dextran measurements were performed in duplicate by fluorimeter (excitation, 485 nm; emission, 535 nm; VICTOR^3TM^, PerkinElmer, Zaventem, Belgium). Serial dilutions of FITC-dextran in PBS were used to calculate a standard curve.

### Sequencing and Processing of Bacterial 16S rRNA

Faecal pellets were collected longitudinally from mice at both 3 and 8 weeks of age and were stored within 2 hours after sampling at −80°C until processing. The data set comprised 38-paired samples, 18 originated from P mice and 20 from NP mice. Nineteen samples (9 P mice and 10 NP mice) belonged to mice receiving NC, while 10 (5 P mice and 5 NP mice) and 9 (4 P and 5 NP) samples belonged to mice receiving the 400 and 800 IU/day vitamin D-supplemented diet, respectively.

Bacterial genomic DNA was extracted from faecal pellets using the Qiagen QIAamp DNA Stool Mini Kit following manufacturer’s instructions (Qiagen Benelux BV, Antwerp, Belgium). Samples for paired-end Illumina MiSeq (the VIB Nucleomics core laboratory, Leuven, Belgium) were constructed using a two-step PCR amplicon approach targeting the V4 region of the 16S rRNA gene as described (32). The 16S rRNA read de-multiplexing was performed using LotuS pipeline v1.62.1 (33). Data pre-processing was performed using DADA2 v.1.14.1 (34), including trimming, quality control, merging of sequencing pairs and taxonomy assignment with the Silva classifier v1.32 (35), using the default parameters. Genera appearing in less than 50% of each dietary or outcome group were excluded for the differential microbiota abundance analyses. Samples were variance stabilization (VS) transformed using the *DESeq2* package (36) in R.

### Statistical Analysis

Data were plotted as mean ± SEM and statistics calculated with GraphPad Prism 8 software (GraphPad Software, La Jolla, CA). Diabetes incidence was evaluated by Kaplan–Meier survival analysis with Mantel-Cox log-rank test. For all other data derived from mice receiving NC or vitamin D-supplemented diets throughout life, differences were estimated by a Student’s two–tailed t test or Mann–Whitney U test if the data did not assume Gaussian distribution. However, when two or more groups were compared, normally distributed data sets were analysed by one–way ANOVA with Bonferroni’s multiple-comparison test, whereas the Kruskal–Wallis test with subsequent Dunn’s multiple-comparison test was used for non-normally distributed data.

Gut microbiota data were analysed by R statistical software using the packages vegan v.2.5-7 (37), phyloseq v1.30.0 (38), FSA v.0.9.1 (39), and stats v.3.6.3 (40). Genera alpha-diversity indices (Shannon diversity and Chao1 richness) were calculated using phyloseq. Due to small sample size, Mann–Whitney U or Kruskal–Wallis test were used to test the differences in microbial taxa and alpha-diversity indices. Only after the Kruskal-Wallis test, we performed a *post-hoc* Dunn’s test, to determine which diets were driving the differences in the corresponding features. The contribution of metadata variables on genus-level microbiota community variation was determined using multivariate distance-based redundancy analysis (dbRDA) using the Bray-Curtis dissimilarity index as implemented in the R package vegan. Microbiome inter-individual variation was visualized by canonical correspondence analysis (CCA) on the genus-level VS transformed abundance matrix using the ggplot2 v.3.3.5 R package (41). P values ≤ 0.05 were considered significant (* ≤ 0.05; ** ≤ 0.01; *** ≤ 0.001; **** ≤ 0.0001).

## Results

### Daily Vitamin D Supplementation With 800 IU, But Not 400 IU, Delayed Disease Onset and Reduced T1D Incidence in NOD Mice

Few data exist on circulating vitamin D concentrations needed to restore immune tolerance in autoimmune-prone subjects. Here, we studied the effect of two doses (400 and 800 IU) of daily lifelong dietary vitamin D supplementation on T1D development in the NOD mouse model and evaluated whether serum vitamin D concentrations correlated with disease outcome.

**Figure 1:**
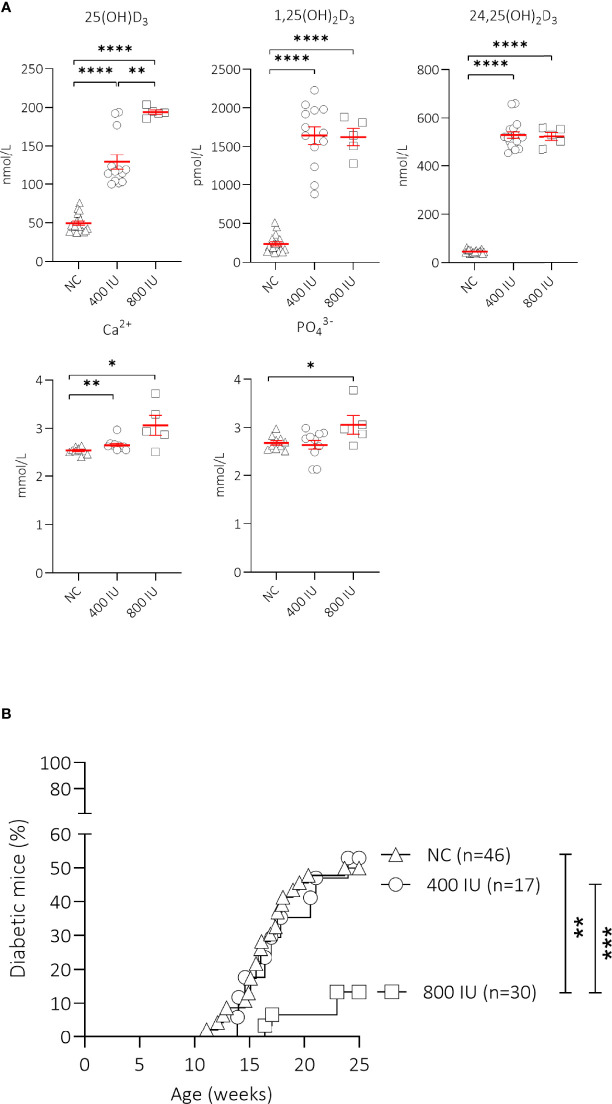
Effect of vitamin D substitution regimen on serum metabolites and type 1 diabetes (T1D) incidence. NOD mice were fed normal chow (NC) or two different doses of vitamin D-supplemented (400 or 800 IU/day) diet from 3 until 25 weeks of age. Serum levels of 25(OH)D_3_, 1,25(OH)_2_D_3_, 24,25(OH)_2_D_3_, calcium, and phosphate are shown for NOD mice of 8 weeks of age (5 weeks on the respective diets). Symbols represent individual mice (N = 5-18), and the line reflects the group mean with SEM. **(A)** Kaplan-Mayer survival curves depict T1D incidence over time. Mice (N = 17-46) with two consecutive measurements of blood glucose values >200 mg/dL were considered diabetic. **(B)**. *P ≤ 0.05; **P ≤ 0.01; ***P ≤ 0.001; ****P ≤ 0.0001.

We first determined the effects of oral vitamin D supplementation on circulating vitamin D metabolites 5 weeks upon dietary intervention (at 8 weeks of age) (**Figure 1A**). A 2.6- and 3.9-fold increase in serum concentrations of 25(OH)D_3_, the major circulating form of vitamin D, was recorded in mice receiving the 400 (129.0 ± 9.6 nmol/L, P ≤ 0.0001) or 800 (193.9 ± 2.9 nmol/L; P ≤ 0.001) IU/day vitamin D-supplemented diet respectively, compared to mice receiving NC (49.5 ± 2.8 nmol/L). Serum values of 25(OH)D_3_ further increased 1.5-fold with the 800 IU/day vitamin D-supplemented diet compared to values obtained under the 400 IU/d vitamin D-supplemented diet. The amount of circulating 1,25(OH)_2_D_3_, the active form of vitamin D, is strictly regulated in a renal negative feedback loop and is independent of circulating 25(OH)D_3_. We observed that serum concentrations of 1,25(OH)_2_D_3_ increased 6.9- and 6.8-fold in mice receiving a 400 (1,644.3 ± 113.6 pmol/L; P ≤ 0.0001) or 800 (1,624.0 ± 109.5 pmol/L; P ≤ 0.0001) IU/day vitamin D-supplemented diet respectively, compared to mice receiving NC (237.1 ± 26.4 pmol/L). Serum 24,25(OH)_2_D_3_ concentrations serve as an indicator of vitamin D catabolic status and depend on the amount of 25(OH)D_3_. We found that serum 24,25(OH)_2_D_3_ concentrations increased 11.5- and 11.4-fold in mice receiving a 400 (529.6 ± 13.2 nmol/L; P ≤ 0.0001) or 800 (522.2 ± 16.4 nmol/L; P ≤ 0.0001) IU/day vitamin D-supplemented diet respectively, compared to mice receiving NC (45.9 ± 1.7 nmol/L).

High vitamin D concentrations may cause hypercalcemia and hyperphosphatemia, which can negatively affect bone and kidney health. Serum calcium values were 1.08- and 1.24-fold higher in mice receiving the 400 (2.65 ± 0.04 mmol/L; P ≤ 0.01) or 800 (3.06 ± 0.21 mmol/L; P ≤ 0.05) IU/day vitamin D-supplemented diet compared to mice receiving NC (2.53 ± 0.02 mmol/L)(**Figure 1A**). Serum phosphate levels were also higher in mice receiving the 800 (3.06 ± 0.19 mmol/L; P ≤ 0.05), but not in those receiving the 400 (2.64 ± 0.10 mmol/L; P = NS) IU/day vitamin D-supplemented diet compared to mice receiving NC (2.69 ± 0.05 mmol/L), indicating signs of moderate hypercalcemia and -phosphatemia.

Interestingly, only the 800 IU/day vitamin D-supplemented diet was able to significantly reduce T1D development in female NOD mice compared to mice receiving NC (13 vs. 50% at 25 weeks of age; P ≤ 0.01), with a delay in disease onset of 5 weeks compared to mice receiving NC (**Figure 1B**). T1D onset and incidence in mice receiving the 400 IU/day vitamin D-supplemented diet was comparable to values obtained in mice receiving NC (disease onset at 14 vs. 12 weeks of age, T1D incidence 53 vs. 50% respectively, P = NS).

### Only High-Dose Vitamin D Supplementation Maintained Increased Frequencies of Splenic FoxP3^+^ Treg Cells

Treg cells are the central component for maintaining peripheral tolerance. Here, we studied FoxP3^+^ Treg cells in spleen, and draining lymph nodes (of the pancreas and gut) at both 8 and 25 weeks of age using multi-colour flow cytometry.

**Figure 2:**
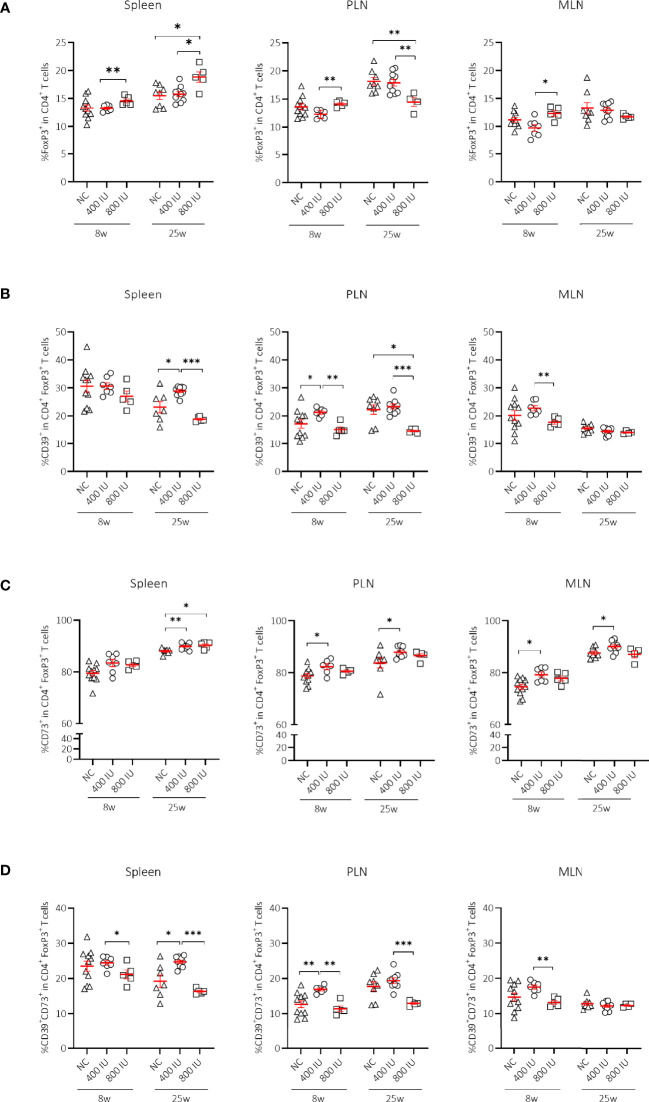
Effect of vitamin D substitution regimen on frequency and functionality of Treg cells. Frequency of FoxP3^+^ T cells within CD4^+^ T-cell gate **(A)** and their functionality as expressed by the frequency of CD39^+^ **(B)**, CD73^+^ **(C)** or CD39^+^CD73^+^ T cells **(D)** within FoxP3^+^CD4^+^ T-cell gate are shown at both 8 and 25 weeks of age in spleen, pancreatic draining lymph nodes (PLN), and mesenteric draining lymph nodes (MLN). Female NOD mice were fed normal chow (NC) or two different doses of vitamin D-supplemented (400 or 800 IU/day) diet from 3 until 25 weeks of age (lifelong). Symbols (N = 4-11) represent individual mice, and line reflects group mean with SEM. *P ≤ 0.05; **P ≤ 0.01; ***P ≤ 0.001.

The frequencies of FoxP3^+^ Treg cells in all studied immune organs were similar between mice receiving the 400 IU/day vitamin D-supplemented diet compared to those on NC at 8 and 25 weeks of age. However, the percentages of FoxP3^+^ Treg cells in mice receiving the 800 IU/day vitamin D-supplemented diet were increased in the spleen, PLN, and MLN, compared to mice receiving the 400 IU/day vitamin D-supplemented diet at 8 weeks of age (**Figure 2A**). These values further augmented however, only in the spleen of mice receiving the 800 IU/day vitamin D-supplemented diet compared to mice receiving either the 400 IU/day vitamin D-supplemented diet or NC at 25 weeks of age (**Figure 2A**).

Although expression of the IL-2Rα chain (CD25) by CD4^+^ T cells follows activation, its expression by CD4^+^ T cells is utilised extensively as a marker to classify Treg cells. The percentages of CD25^+^FoxP3^+^ Treg cells were significantly increased in the spleen of mice receiving the 800 IU/day vitamin D-supplemented diet compared to mice receiving a 400 IU/d vitamin D-supplemented diet at 8 and 25 weeks of age (**Supplementary Figure 3A**). Moreover, the frequencies of the CD25^–^FoxP3^+^ Treg cell subset were also expanded however, only by the 800 IU/d vitamin D diet in the spleen, PLN and MLN at 25 weeks of age (**Supplementary Figure 3B**).

### FoxP3^+^ Treg Cell Expansion Upon High-Dose Vitamin D Supplementation Was Associated With Co-Expression of the Ecto-5’-Nucleotidase CD73 in the Spleen

Several mechanisms have been proposed as to how Treg cells exert their suppressive function including cell-to-cell contact and the release of soluble mediators. Recent data point to CD39 and CD73 as novel markers of FoxP3^+^ Treg cells that subsequently degrade the extracellular ATP pool and catalyse the formation of adenosine, which has the ability to dampen aberrant immune reactions (42). Surprisingly, the frequency of CD39^+^ and of CD39^+^CD73^+^ T cells within the CD4^+^Foxp3^+^ T-cell gate was reduced in the spleen, MLN, and PLN of mice receiving the 800 IU/day vitamin D-supplemented diet compared to mice receiving the 400 IU/day vitamin D-supplemented diet or NC. This observation was present at 8 weeks of age, but more pronounced at 25 weeks of age (**Figures 2B, D**). On the other hand, the CD73^+^ T-cell population within the CD4^+^FoxP3^+^ T-cell gate was significantly enlarged in the spleen of mice receiving the 800 IU/day vitamin D-supplemented diet compared to mice receiving the 400 IU/d vitamin D-supplemented diet or NC at 25 weeks (**Figure 2C**), indicating that under a high-dose vitamin D substitution regimen FoxP3^+^ T cells in the periphery may function in a CD73-dependent manner.

### High-Dose Vitamin D Supplementation Increased Frequencies of IL-10-Secreting CD4^+^ T Cells

**Figure 3:**
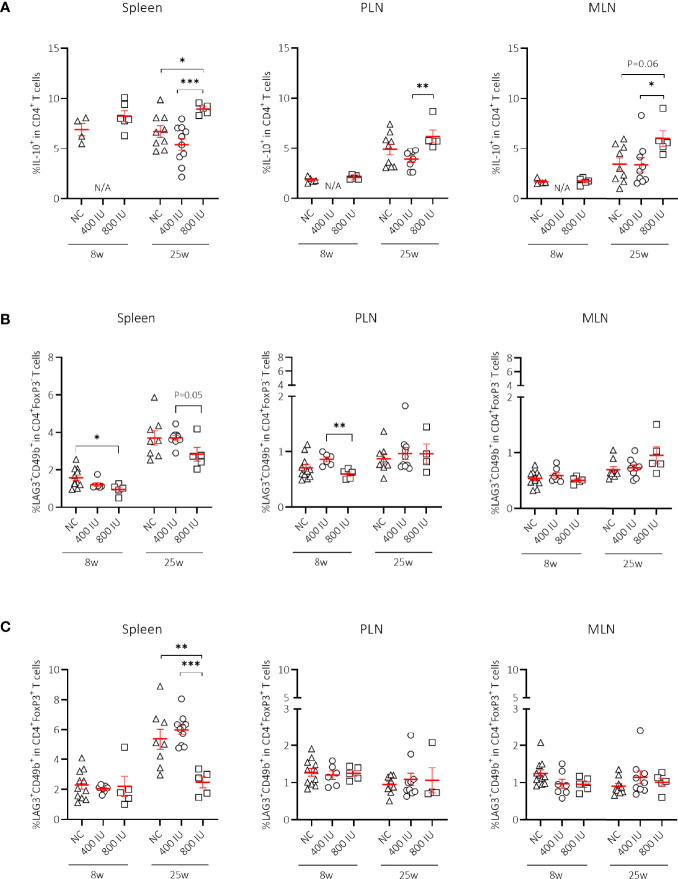
Effect of vitamin D substitution regimen on frequency of IL-10-secreting CD4^+^ T cells. Frequency of IL-10-secreting CD4^+^ T cells within CD4^+^ T-cell gate **(A)** and frequency of Tr1 cells as defined as LAG3^+^CD49b^+^ within the CD4^+^FoxP3^–^ **(B)**, and CD4^+^FoxP3^+^ T-cell gate **(C)** are shown at both 8 and 25 weeks of age in spleen, pancreatic draining lymph nodes (PLN), and mesenteric draining lymph nodes (MLN). Female NOD mice were fed normal chow (NC) or two different doses of vitamin D-supplemented (400 or 800 IU/day) diet from 3 until 25 weeks of age (lifelong). Symbols (N = 4-11) represent individual mice, and the line reflects the group mean with SEM. *P ≤ 0.05; **P ≤ 0.01; ***P ≤ 0.001.

Apart from FoxP3^+^ Treg cells, IL-10-secreting T cells may constitute a supplementary mechanism responsible for peripheral tolerance. Different IL-10-secreting T cells have been described such as Tr1 cells (3), Treg cells expressing the latency-associated peptide (LAP), but also IL-10-secreting CD4^+^CD25^hi^CD127^–^FoxP3^–^ T cells induced by vitamin D in healthy and T1D individuals (17). Supplementing NOD mice with the 800 IU/day vitamin D diet increased the percentages of CD4^+^IL-10^+^ T cells within the spleen, PLN, and MLN compared to values obtained in mice on the 400 IU/day vitamin D diet or NC at 25 weeks of age (**Figure 3A**). We further explored whether Tr1 cells, which are characterized by co-expression of CD49b and LAG3, high secretion of IL-10, and lack of FoxP3 expression, were responsible for the vitamin D-induced rise in IL-10^+^CD4^+^ T cells (3). However, we did not find an expansion of Tr1 cells in any of the studied immune organs by vitamin D at 8 nor at 25 weeks of age (**Figure 3B**). Recent data based on the use of an IL-10-GFP/FoxP3-RFP dual reporter transgenic model demonstrated that co-expression of CD49b and LAG3 was not restricted to the FoxP3^–^ Tr1 cells, but was also observed in Foxp3^+^ Treg cells (43). Percentages of Foxp3^+^ Tr1 cells were not increased but on the contrary diminished in the spleen of NOD mice upon the 800 IU/day vitamin D-supplemented diet compared to the other groups at 25 weeks of age (**Figure 3C**).

### High-Dose Vitamin D Supplementation Did Not Correct Gut Leakiness in NOD Mice

**Figure 4:**
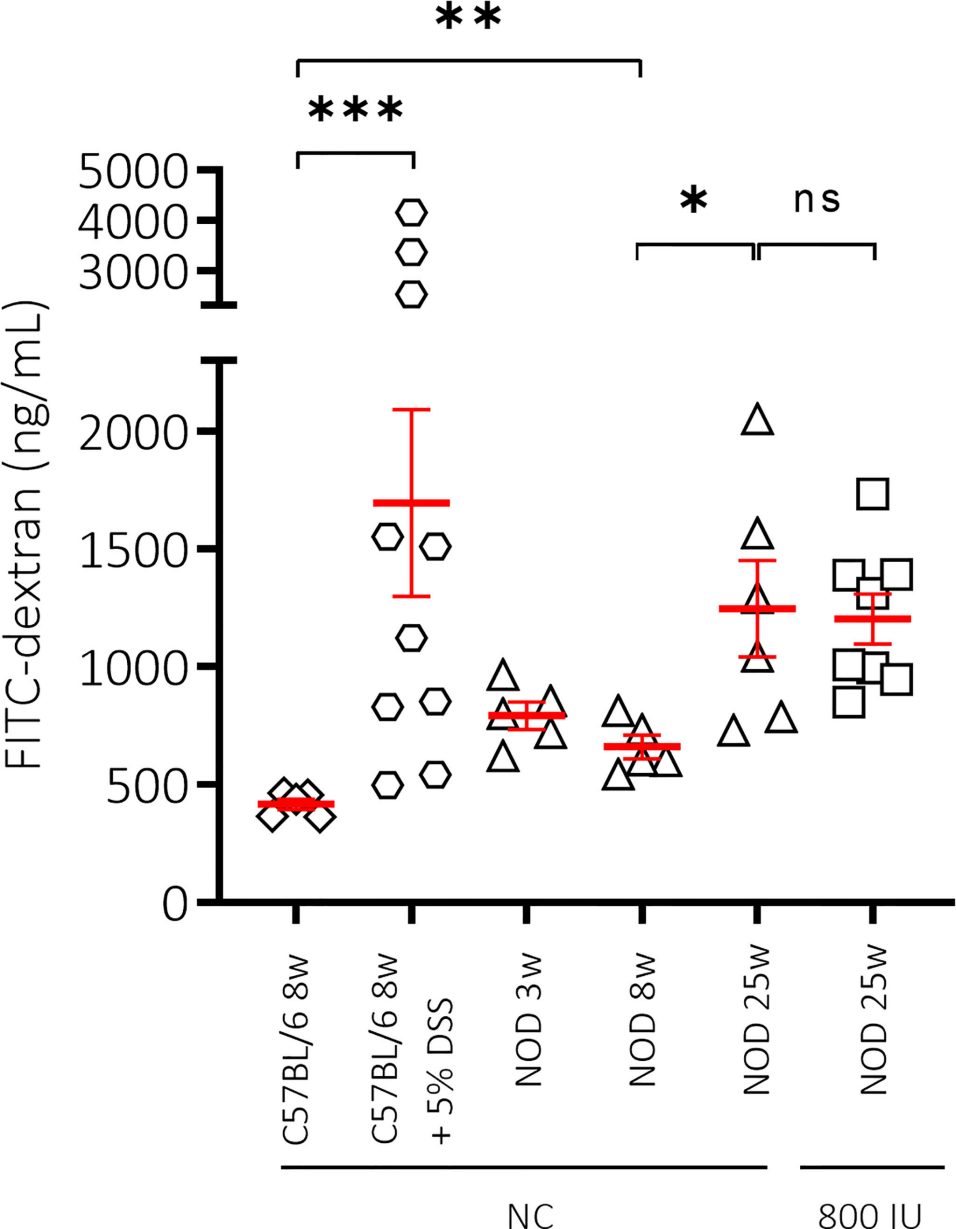
Effect of mouse strain, age and vitamin D substitution regimen on intestinal permeability. Concentration of FITC-dextran as measurement for intestinal permeability is shown for female C57BL/6 mice at 8 weeks of age in addition to female NOD mice at increasing ages (3, 8, and 25 weeks of age). Mice received either normal chow (NC) or the 800 IU/day vitamin D-supplemented diet from 3 until 25 weeks of age. To validate our technique, dextran sulfate sodium (DSS) was added at 5% in drinking water *ad libitum* for 8 days to 8-week-old C57BL/6 mice. Symbols (N = 3-10) represent individual mice, and the line reflects the group mean with SEM. *P ≤ 0.05; **P ≤ 0.01; ***P ≤ 0.001. NS means non significant.

Loss of gut epithelial barrier function has been linked to the activation of autoreactive T cells and T1D onset (44). Moreover, vitamin D was demonstrated to maintain gut barrier integrity (45). Here, we evaluated the effect of the highest dose (800 IU/day) of the vitamin D diet on gut epithelial barrier function in non-diabetic female NOD mice. As a negative and positive control, we included female healthy C57BL/6 as well as DSS-treated C57BL/6 mice of 8 weeks of age, respectively. We observed that NOD mice at 8 weeks of age already had a leaky gut compared to age-matched healthy C57BL/6 mice as exemplified by increased serum levels of FITC-labelled dextran (P ≤ 0.01; **Figure 4**). We also demonstrated that gut leakiness further increased with age in the NOD mice, while supplementation with 800 IU/day of regular vitamin D did not correct the breakage of the gut barrier integrity (P = NS; **Figure 4**).

### Microbiota Diversity and Richness Increases With Aging in NOD Mice, Especially in Mice Not Progressing to Diabetes

Previous publications have reported that the gut microbiome from NOD mice undergoes significant modifications between 3 and 8 weeks of age (46). Here, we assembled a *phyloseq* object consisting of 105 genera and 10 phyla on 38 paired samples. Phyla or genera not present in at least 50% of each experimental group were filtered-out, leaving 7 phyla and 35 genera.

**Figure 5:**
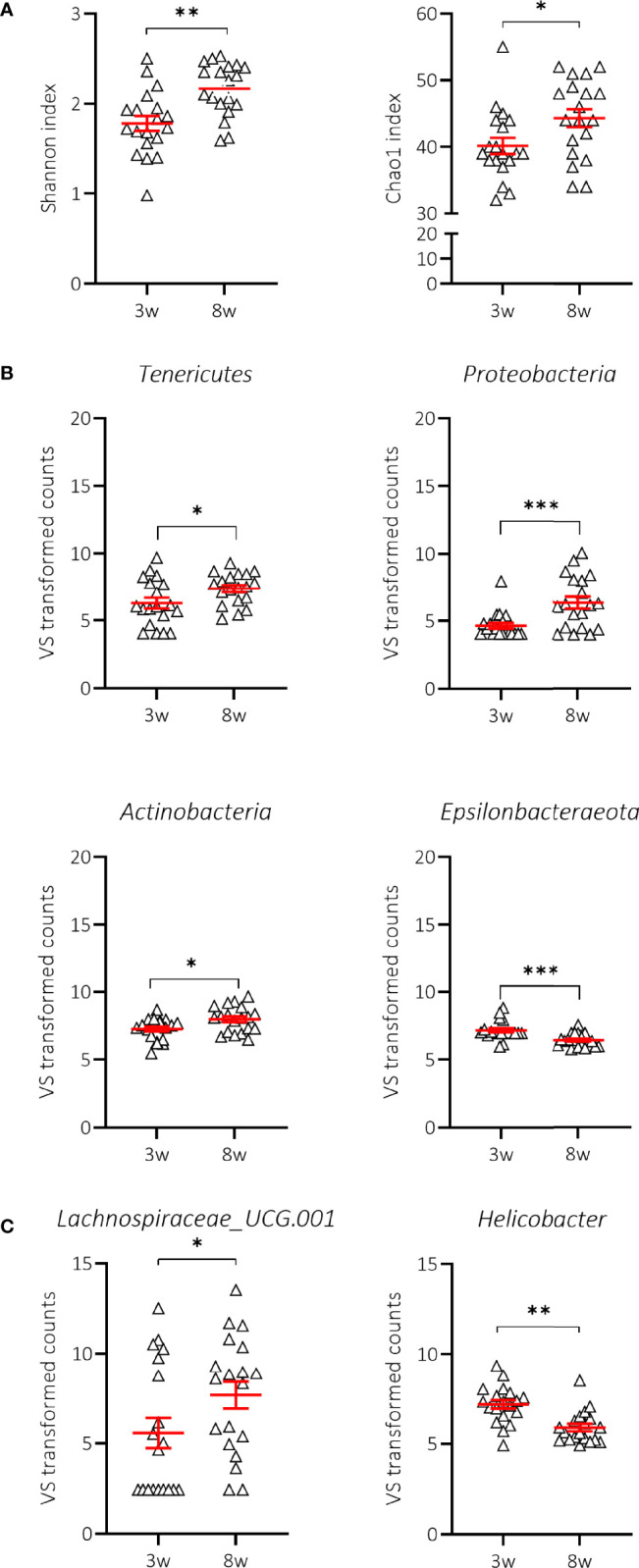
Change in alpha-diversity and taxa abundances of NOD mice receiving normal chow (NC). Changes in Shannon and Chao1 indices **(A)**, phyla **(B)** and genera **(C)** are shown for female NOD mice receiving normal chow (NC) (N = 38 paired samples). Symbols (N = 9-19) represent individual mice, and the line reflects the group mean with SEM. *P ≤ 0.05; **P ≤ 0.01; ***P ≤ 0.001.

We first demonstrated that the composition of the gut microbiome at 3 weeks of age (weaning) in mice receiving NC was indeed significantly different from that at 8 weeks of age, a time when all mice were still normoglycaemic. We observed that the Shannon’s alpha-diversity index (genera diversity in terms of genera richness and relative abundance) and the Chao1 index (genera richness) significantly increased between 3 and 8 weeks of age in NOD mice receiving NC (**Figure 5A**; **Supplementary Table S1**), indicating increased taxonomic complexity of bacterial communities. At the phylum level, NOD mice receiving NC showed a significant difference over time in 4 out of 7 tested phyla with an increase in *Tenericutes*, *Proteobacteria*, and *Actinobacteria*, in addition to a decrease in *Epsilonbacteraeota* (**Figure 5B**; **Supplementary Table S2**), but overall we did not find a significant change in the *Bacteroidetes*:*Firmicutes* ratio between 3 and 8 weeks of age in NOD mice receiving NC (data not shown). At the genus level, NOD mice receiving NC showed an increase in the abundance of *Lachnospiraceae UCG-001* and a decrease in *Helicobacter* between 3 and 8 weeks of age (**Figure 5C**; **Supplementary Table S3**).

**Figure 6:**
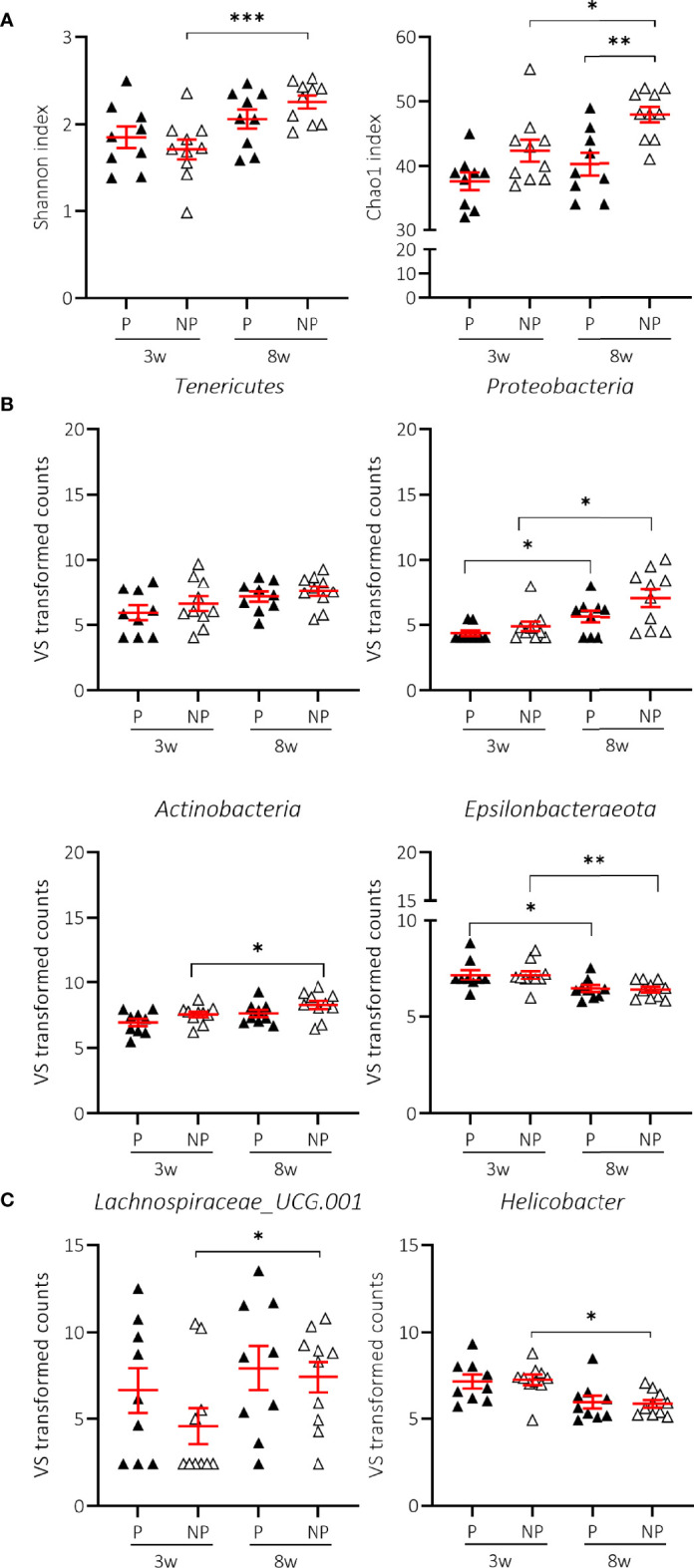
Change in alpha-diversity and taxa abundances of T1D progressor (P) and non-progressor (NP) NOD mice receiving normal chow (NC). Changes in Shannon and Chao1 indices **(A)**, phyla **(B)**, and genera **(C)** are shown for female NOD mice receiving NC and progressing (P) (N = 18 paired samples) or not progressing towards T1D (NP) (N = 20 paired samples). Symbols (N = 4-10) represent individual mice, and the line reflects the group mean with SEM. *P ≤ 0.05; **P ≤ 0.01; ***P ≤ 0.001.

We next examined whether there were characteristics of the microbial community that could discriminate the T1D status. We assessed the Shannon and Chao1 indices across time in P and NP mice receiving NC. Interestingly, the greater diversity and richness of the different taxa over time was only significant in NP mice receiving NC (**Figure 6A**; **Supplementary Table S1**). The increased alpha-diversity in NP went along with a significant increase in *Tenericutes* and a decrease in *Epsilonbacteraeota* phyla. *Proteobacteria* and *Actinobacteria* were also increased, although this was not statistically significant (**Figure 6B**; **Supplementary Table S2**). Moreover, the *Bacteroidetes*:*Firmicutes* ratio was not related to disease outcome (data not shown). At the genus level, we demonstrated that the differential abundance observed over time in *Lachnospiraceae UCG-001* and *Helicobacter* was only significant in the NP mice receiving NC (**Figure 6C**; **Supplementary Table S3**). We also identified a drop in alpha-diversity in faecal samples collected at 8 weeks of age of P compared to NP mice receiving NC (**Figure 6A**; **Supplementary Table S2**). Intriguingly, this divergence in alpha-diversity occurred at a time before the P mice presented with clinical disease.

### Both Diet and Disease Outcome Influence the Microbial Community Composition in NOD Mice

**Figure 7:**
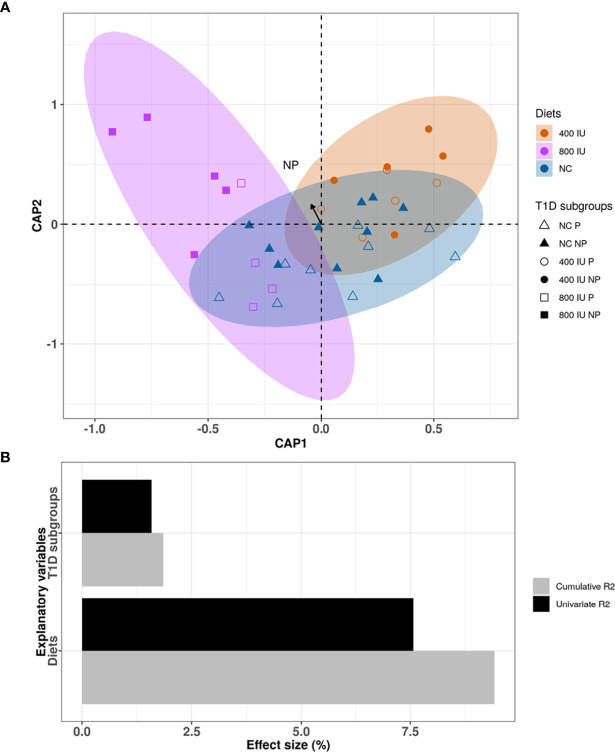
Differences in microbial community composition between NOD mice receiving normal chow (NC), 400 IU/day or 800 IU/day vitamin D-supplemented diet and T1D subgroups at 8 weeks of age. Canonical analysis of principal coordinates (CAP) visualization of inter-mice differences in the microbiome profiles of the NOD mice cohort at 8 weeks of age (N = 38 biologically independent samples, data points coloured by diet and disease outcome subgroups denoted by shape). The figure is stratified by dietary subgroups depicting the differences in microbial composition of the mice receiving NC (N = 38 paired samples, blue), 400 (N = 20 paired samples, orange) or 800 IU/day (N = 18 paired samples, purple) vitamin D-supplemented diets at 8 weeks of age. The arrow points to the centroid of the T1D NP subgroup **(A)**. Bar plot representation of the explanatory power of the diet and disease outcome on the NOD microbiota compositional variation in a single variable model (univariate effect size [R2]) or combined in a multivariate model (cumulative R2) **(B)**. Statistical significance was calculated by distance-based redundancy analysis (Bray-Curtis dissimilarity index).

Although the 400 and 800 IU/day vitamin D-supplemented diets did not significantly modify the increased alpha-diversity nor the different abundance in specific phyla observed over time in NOD mice receiving NC, a multivariate dbRDA analysis revealed that diet in addition to disease outcome induced significant changes in the composition of the gut microbiome at 8 weeks of age at the genus level (multivariate dbRDA, total adjusted *R* ^2^ = 9.4%; diet P = 0.001; individual adjusted *R* ^2^ = 7.57%. disease outcome P = 0.046. individual adjusted *R* ^2^ = 1.58%; N = 38)(**Figures 7A, B**). Furthermore, a *post hoc* comparison of the diets revealed that the 800 IU/day vitamin D diet significantly deviated in microbial community composition from mice receiving the 400 IU/day vitamin D-supplemented diet or NC (univariate dbRDA, adjusted R2 = 2.34%; P  = 0.012; N = 28 and R2 = 7.57%; P  = 0.001; N = 19; respectively (**Figure 7B**).

### High-Dose Vitamin D Supplementation Altered the Abundance of *Ruminiclostridium_9* and *Marvinbryantia* in Faecal Samples of 8-Week-Old NOD Mice, Irrespective of Disease Outcome

**Figure 8:**
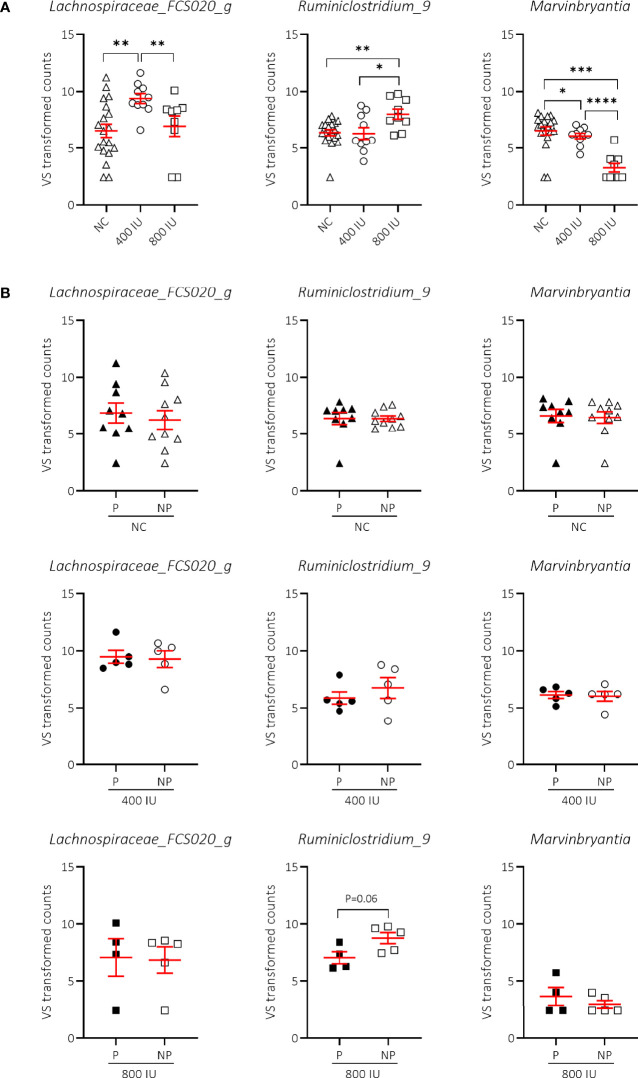
Difference in genera abundances between diets and T1D subgroups at 8 weeks of age. Differences in genera abundances between diets at 8 weeks of age are shown for female NOD mice receiving normal chow (NC), 400 or 800 IU/day vitamin D-supplemented diets **(A)**. Differences in genera abundance for the corresponding diets shown in **(A)** are separated based on disease outcome **(B)**. P = progressor mice, NP = non-progressor mice. Symbols (N = 4-19) represent individual mice, and the line reflects the group mean with SEM. *P ≤ 0.05; **P ≤ 0.01; ***P ≤ 0.001; ****P ≤ 0.0001.

We demonstrated that vitamin D supplementation correlated with non-redundant changes in the NOD microbial community composition at 8 weeks of age (**Figure 7**). First, we noticed that the vitamin D-supplemented diet had no influence on the increased abundance of *Lachnospiraceae UCG-001* and *Helicobacter* identified in faecal samples of NOD mice at 8 weeks of age receiving NC (data not shown). On the other hand, we identified two bacterial genera that discriminated the 800 IU/day vitamin D group including *Ruminiclostridium_9* and *Marvinbryantia* from both the 400 IU/day vitamin D and NC groups at 8 weeks of age (**Figure 8A**; **Supplementary Table S3**). The abundance of the *Ruminiclostrium_9* and *Marvinbryantia* significantly increased or decreased, respectively with accumulating doses of vitamin D (**Figure 8A**; **Supplementary Table S3**). The abundance of the genus *Lachnospiraceae_FCS020_group* increased in faecal samples of mice receiving the 400 IU/day vitamin D-supplemented diet compared to both NC and 800 IU/day vitamin D-supplemented diet (**Figure 8A**). The diet-induced changes in the gut microbial community in NOD mice at 8 weeks of age did not correspond to disease outcome, although faecal samples of NP mice on the 800 IU/day vitamin D diet had a tendency for an increased abundance in the genus *Ruminiclostridium_9* (**Figure 8B**; **Supplementary Table S3**).

## Discussion

In the past, our team demonstrated that an 800 IU/day vitamin D-supplemented diet safely reduced T1D incidence in autoimmune diabetes-prone NOD mice, when given lifelong (17). While the scientific community is convinced that vitamin D deficiency (<25 nmol/L 25(OH)D_3_ concentrations) should be avoided to prevent skeletal defects (47), there is less consensus on which prescriptions of oral vitamin D supplements or circulating 25(OH)D_3_ concentrations are needed to interfere with the initiation and progression of inflammatory and autoimmune pathologies including T1D. Our previous study indicated that serum 25(OH)D_3_ concentrations reaching a mean value of 290 nmol/L at the end of the study, in 35-week-old NOD mice, was linked to T1D protection. These concentrations are way beyond the 50 and 75 nmol/L that are respectively recommended by the Institute of Medicine (IOM) (48) and the Endocrine Society Task Force (20) for maximum bone health for children and adults. On the other hand, many authorities proclaim that optimal serum 25(OH)D_3_ concentrations for all health issues should be above 100 nmol/L (49). Behind this background, the Endocrine Society advocated an ideal range for circulating 25(OH)D_3_ between 100 and 150 nmol/L, and judged a level up to 250 nmol/L as risk-free (20, 50, 51).

Hence, we studied whether a lower dose, 400 IU/day, of dietary vitamin D supplements would be equally effective in delaying and preventing T1D onset in NOD mice. Moreover, by studying not only the peripheral and local (PLN) immune systems, also the effects of vitamin D on different characteristics of the gut (i.e., MLN, intestinal barrier function, and intestinal microbiota composition), we hoped to acquire insights into the mechanisms by which vitamin D elicits disease protection in NOD mice. We demonstrated that only the 800, and not the 400, IU/day vitamin D-supplemented diet could significantly delay and prevent T1D development in NOD mice by 25 weeks of age. We also found that at 5 weeks on the diet (at 8 weeks of age), serum 25(OH)D_3_, but not 1,25(OH)_2_D_3_ and 24,25(OH)_2_D_3_, concentrations positively correlated with T1D outcome later in life. Although 1,25(OH)_2_D_3_ is the active (hormonal) form of vitamin D, its circulating values are 1,000-fold less compared to 25(OH)D_3_ values and it has a short half-life (4-6 hours), limiting its utility as biomarker for vitamin D status. Both 25(OH)D_3_ and 1,25(OH)_2_D_3_ undergo further metabolism, primarily by renal 24-hydroxylase, to generate 24,25(OH)_2_D_3_ (52). The production of 24,25(OH)_2_D_3_, the major circulating catabolite of vitamin D, is only modestly affected by vitamin D supplementation (53), and its physiological role remains elusive. While we observed that a particular threshold value for circulating 25(OH)D_3_, above 150 nmol/L, was linked to T1D protection and accompanied by moderate hypercalcemia and -phosphatemia in mice, hard evidence from other disease models is currently lacking to make strong recommendations on the optimal vitamin D concentrations needed to avoid development of cancer, infections, metabolic and autoimmune diseases in humans.

Amongst the many reported extra-skeletal effects of vitamin D, its capability to modulate both the innate and adaptive systems has obtained significant attention. It is traditionally believed that bioactive 1,25(OH)_2_D_3_ functions directly *via* its receptor, being present on almost all cells of the immune system (14). Many immune cells also possess vitamin D metabolizing enzymes which can affect the autocrine/paracrine vitamin D system and subsequently promote or abrogate response of 25(OH)D_3_. In this context, vitamin D can promote a shift from an inflammatory T helper (Th)1 towards a tolerogenic response *via* the induction or expansion of Th2 cells and Treg cells (13, 54). While some researchers observed only increases in the frequency of Treg cells (55), others reported improved suppressive capacity of Treg cells without alterations in their abundance (56). Although not completely straightforward in this study, we did detect a persistent increase in the percentages of FoxP3^+^ Treg cells in the spleen of the 800 IU/day vitamin D-supplemented mice at 25 weeks of age compared to the other dietary groups. Treg cells can exert their suppressive function by several mechanisms, including the release of immunosuppressive cytokines such as IL-10 and TGF-β, or *via* cellular communication (57). Active vitamin D has been shown to instruct CD4^+^ T cells to convert into CD4^+^CD25^hi^IL-10^+^ Treg cells. However, there is currently no consensus on whether these cells also express the lineage-specifying transcription factor *FoxP3* (58, 59). In this study, we also observed an increase in the frequencies of IL-10-producing CD4^+^ T cells in all studied immune organs of mice at 25 weeks of age receiving the 800 IU/day vitamin D-supplemented diet but this increase was not due to an expansion of FoxP3^–^, nor of FoxP3^+^, Tr1 cells, co-expressing LAG3 and CD49b. A recent transcriptomic analysis substantiated our results that vitamin D does not enrich for genes in the Tr1 cell signature. On the other hand, they found that vitamin D, through autocrine/paracrine production, can generate IL-10-producing CD4^+^ T cells *via* IL-6 and STAT3 signalling (60).

A few remarkable reports prompted us to study whether vitamin D could influence the frequency of FoxP3^+^ Treg cells endowed with the expression of the cell surface ecto-enzymes CD39 and CD73 that subsequently regulate pericellular adenosine accumulation from extracellular nucleotide catabolism (61, 62). Adenosine has been shown to suppress Teff functions by binding to a number of adenosine receptors (i.e., A1R, A2AR, A2BR, and A3R) that are expressed in some immune cell subsets and endothelial cells (42, 63). While vitamin D has been shown to upregulate the expression of CD39 and CD73 on CD4^+^FoxP3^+^ and/or CD4^+^Foxp3^–^ T cells, expressing the late-stage Treg activation markers glycoprotein A repetitions predominant (GARP) and LAP, in addition to neuropilin-1 (61, 62), we did not study the expression level (i.e., mean fluorescent intensity) of CD39 and CD73 on FoxP3^+^ Treg cells. However, we did identify an 800 IU/day vitamin D-induced increase in the abundance of CD73^+^FoxP3^+^ Treg cells in mice at 25 weeks of age, but only in the systemic circulation. CD73 has been shown to be critical in mediating many immunosuppressive features of T cells. Whether CD73-expressing FoxpP3^+^ T cells exert their function in T1D development by patrolling the systemic circulation and subsequently suppressing Teff activation and/or limiting Teff migration to the PLN and pancreas warrants further investigation. Intriguing observations in the T1D field indicate that CD73^+^CD4^+^ T cells can inhibit islet-reactive T-cell proliferation by the production of the anti-inflammatory cytokine TGF-β, yet not of IL-10 (64).

As mentioned before, intestinal dysbiosis, low-grade intestinal inflammation, and intestinal barrier dysfunction at an early age, allowing increased antigen (i.e., dietary or microbial) trafficking, could trigger T1D initiation *via* the activation of diabetogenic T cells in the intestinal mucosa (44, 65). Alternatively, loss of intestinal barrier function could allow the release of bacterial pathogens with molecular mimicry to islet antigens in the periphery, which could directly damage insulin-producing β cells or activate islet-reactive T cells within the PLN and pancreas (8). We confirmed by a FITC-dextran assay that NOD mice of 3 weeks of age (at weaning) already have augmented gut permeability, a time point before the development of insulitis and overt hyperglycaemia, which further increased with age. Although the regimen with 800 IU/d of dietary vitamin D did not correct the intestinal barrier dysfunction in NOD mice by 25 weeks of age, we tested *via* 16S rRNA sequencing whether intestinal dysbiosis could be another gut-related trait leading to T1D initiation and could be modified by vitamin D. An aberrant microbial composition of the gut may result in a distorted maturation of the immune system and increase the vulnerability to immune-mediated diseases. We found that bacterial Chao1 richness, Shannon diversity, and the relative abundance of particular phyla (i.e., *Proteobacteria*, *Tenericutes*, and *Actinobacteria*) increased in NOD mice during aging (from 3 until 8 weeks of age), specifically in NOD mice not further progressing to overt hyperglycaemia. A greater intestinal microbiota diversity is conducive to enrichment of bacterial taxa that can produce bile acids and short chain fatty acids (SCFA) like acetate, butyrate, and propionate, which are beneficial for not only gut barrier function, and intestinal immunomodulation, but also help maintain homeostasis and health during the whole lifespan (66–68). The *Bacteroidetes* phylum is associated with the production of acetate and propionate, while the *Firmicutes* phylum mainly produces butyrate. Here, the ratio *Bacteroidetes to Firmicutes* was not modified in NOD mice over time nor was it related to disease outcome. Conflicting reports have been published with some authors demonstrating a successive decline in *Firmicutes* and increase in *Bacteroidetes* species in the intestinal microbiome of children during the first 6 months of life before T1D development (69), while others did not found any correlation between *Bacteroidetes* abundance and T1D onset (70). Still, our data propose that the phyla *Proteobacteria*, *Tenericutes*, and *Actinobacteria* were associated with T1D protection in NOD mice later in life.

Diet is an important factor in intestinal microbiota shaping (68). Although vitamin D did not alter the bacterial richness, diversity, and phyla abundance in NOD mice over time, a multivariate dbRDA analysis revealed a significant association between community composition and vitamin D supplementation at the genus level. The 800 IU/day vitamin D-supplemented group had a higher relative abundance of the genus *Ruminiclostridium_9* and a lower relative abundance of the genus *Marvinbryantia* compared to the 400 IU/day vitamin D-supplemented and NC groups at 8 weeks of age. We also observed a trend of a higher relative abundance of *Ruminiclostridium_9* in mice not further progressing to T1D. Little is known about the physiological and pathogenic roles of *Ruminiclostridium*_9 and *Marvinbryantia* in the gastrointestinal tract. A recent report found a decrease in *Ruminiclostridium_9* to be associated with exacerbated insulitis in NOD mice (71). Both *Ruminiclostridium_9* and *Marvinbyrantia* belong to the phylum *Firmicutes* and are proposed to be involved in the conversion of primary to secondary bile acids and butyrate production. The beneficial effects of butyrate on local and systemic immunity are well defined. Butyrate can recruit Treg cells in the colon as well as the pancreas (72) and modulate their function, linking them to crosstalk between the intestinal microbiota and immune system. Moreover, butyrate has been observed to increase intestinal VDR expression (73), which could further potentiate the effect of vitamin D. Vitamin D seems to have opposing effects on the abundance of these two genera, necessitating more in depth studies with larger sample size.

In summary, our investigation of the influence of dietary vitamin D supplementation on T1D development revealed that only the higher dose (800 IU/day) could delay disease onset and significantly reduce T1D incidence in NOD mice. In an attempt to characterise the mechanisms of T1D protection elicited by this dosing regimen, we identified a peripheral expansion of FoxP3^+^ Treg cells that may modulate autoimmune inflammation in a CD73-dependent manner. Increased frequencies of IL-10-producing CD4^+^ T cells in various immune organs of mice given the 800 IU/d vitamin D-supplemented diet may constitute a supplementary mechanism in restoring peripheral tolerance. In addition, high-dose vitamin D supplementation and T1D protection were associated with alterations in microbial community composition favouring *Ruminiclostridium_9* and diminishing *Marvinbryantia* at the genus level. The results of this preclinical study are promising, but also warrant more research into the interplay between vitamin D, the gut microbiota, and T1D progression. Indeed, further insights are needed into the specific molecular interactions with the host immune responses and how the gut microbiota may alter T1D development. Additionally, human studies will have to be designed to ensure translatability of our findings. This study sheds further light on the potential of vitamin D as an environmental exposure in delaying and preventing T1D in genetically predisposed individuals. Integration of these findings into our understanding of T1D may help to advance therapeutic efficacy in the new age of preventative treatments.

## Data Availability Statement

The original contributions presented in the study are publicly available. This data can be found here: https://www.ebi.ac.uk/ena/browser/view/PRJEB51877.

## Ethics Statement

All animal experiments were approved by the Ethics Committee of the KU Leuven Animal Care and Use Committee and compiled with Belgian animal protection law under animal experiment license 114/2015.

## Author Contributions

P-JM, JC-L, DC, and DE designed and performed research as well as analysed and discussed the data and wrote the manuscript. LV did experiments and analysed data. LV, AV, and JR offered resources. JR discussed the data. CM and CG conceptualized the research goals, acquired major funding, designed research, analysed and discussed the data, and wrote the manuscript. All authors contributed to the article and approved the submitted version.

## Funding

This work was mainly supported by KU Leuven (C16/18/006) and the Fonds voor Wetenschappelijk Onderzoek Vlaanderen (FWO; DC. [11Y6716N]). This publication was supported by the Open Access Publication Fund of the KU Leuven.

## Conflict of Interest

The authors declare that the research was conducted in the absence of any commercial or financial relationships that could be construed as a potential conflict of interest.

## Publisher’s Note

All claims expressed in this article are solely those of the authors and do not necessarily represent those of their affiliated organizations, or those of the publisher, the editors and the reviewers. Any product that may be evaluated in this article, or claim that may be made by its manufacturer, is not guaranteed or endorsed by the publisher.

## Abbreviations

1,25(OH)_2_D_3_, 1,25-dihydroxyvitamin D_3_; 24,25(OH)_2_D_3_, 24,25-dihydroxyvitamin D3; 25,25-dihydroxyvitamin D_3_; 25(OH)D_3_, 25-hydroxyvitamin D_3_; DSS, dextran sulfate sodium; FoxP3, forkhead box P3; GARP, glycoprotein A repetitions predominant; IL, interleukin; LAG3, lymphocyte-activation gene 3; LAP, latency-associated peptide; LC-MS/MS, liquid chromatography tandem mass spectrometry; MLN, mesenteric lymph nodes; NC, normal chow; NOD, non-obese diabetic; NP, non-progressor; OTU, operational taxonomic unit; P, progressor; PLN, pancreatic lymph nodes; SPF, specific pathogen free; T1D, type 1 diabetes; Teff, effector T cell; Th, T helper cell; Treg, regulatory T cell; VDR, vitamin D receptor.

## References

[1] Richardson SJ Morgan NG. Enteroviral Infections in the Pathogenesis of Type 1 Diabetes: New Insights for Therapeutic Intervention. Curr Opin Pharmacol (2018) 43:11–9. doi: 10.1016/j.coph.2018.07.006 PMC6294842 30064099

[2] Bach JF. Revisiting the Hygiene Hypothesis in the Context of Autoimmunity. Front Immunol (2020) 11:615192. doi: 10.3389/fimmu.2020.615192 33584703 PMC7876226

[3] Huang W Solouki S Carter C Zheng SG August A. Beyond Type 1 Regulatory T Cells: Co-Expression of LAG3 and CD49b in IL-10-Producing T Cell Lineages. Front Immunol (2018) 9:2625. doi: 10.3389/fimmu.2018.02625 30510554 PMC6252342

[4] Dotta F Censini S van Halteren AG Marselli L Masini M Dionisi S. Coxsackie B4 Virus Infection of Beta Cells and Natural Killer Cell Insulitis in Recent-Onset Type 1 Diabetic Patients. Proc Natl Acad Sci U S A (2007) 104(12):5115–20. doi: 10.1073/pnas.0700442104 PMC1829272 17360338

[5] Colli ML Nogueira TC Allagnat F Cunha DA Gurzov EN Cardozo AK. Exposure to the Viral by-Product dsRNA or Coxsackievirus B5 Triggers Pancreatic Beta Cell Apoptosis *via* a Bim / Mcl-1 Imbalance. PloS Pathog (2011) 7(9):e1002267. doi: 10.1371/journal.ppat.1002267 21977009 PMC3178579

[6] Oikarinen M Tauriainen S Oikarinen S Honkanen T Collin P Rantala I. Type 1 Diabetes is Associated With Enterovirus Infection in Gut Mucosa. Diabetes (2012) 61(3):687–91. doi: 10.2337/db11-1157 PMC3282798 22315304

[7] Zhou H Sun L Zhang S Zhao X Gang X Wang G. Evaluating the Causal Role of Gut Microbiota in Type 1 Diabetes and Its Possible Pathogenic Mechanisms. Front Endocrinol (Lausanne) (2020) 11:125. doi: 10.3389/fendo.2020.00125 32265832 PMC7105744

[8] Cole DK Bulek AM Dolton G Schauenberg AJ Szomolay B Rittase W. Hotspot Autoimmune T Cell Receptor Binding Underlies Pathogen and Insulin Peptide Cross-Reactivity. J Clin Invest (2016) 126(9):3626. doi: 10.1172/JCI85679 PMC5004936 27525441

[9] Tai N Peng J Liu F Gulden E Hu Y Zhang X. Microbial Antigen Mimics Activate Diabetogenic CD8 T Cells in NOD Mice. J Exp Med (2016) 213(10):2129–46. doi: 10.1084/jem.20160526 PMC5030808 27621416

[10] García AR Paterou A Lee M Sławiński H Wicker LS Todd JA. Peripheral Tolerance to Insulin is Encoded by Mimicry in the Microbiome. bioRxiv (2019), 881433. doi: 10.1101/2019.12.18.881433

[11] Raab J Giannopoulou EZ Schneider S Warncke K Krasmann M Winkler C. Prevalence of Vitamin D Deficiency in Pre-Type 1 Diabetes and its Association With Disease Progression. Diabetologia (2014) 57(5):902–8. doi: 10.1007/s00125-014-3181-4 24531263

[12] Giulietti A Gysemans C Stoffels K van Etten E Decallonne B Overbergh L. Vitamin D Deficiency in Early Life Accelerates Type 1 Diabetes in non-Obese Diabetic Mice. Diabetologia (2004) 47(3):451–62. doi: 10.1007/s00125-004-1329-3 14758446

[13] Baeke F Korf H Overbergh L Verstuyf A Thorrez L Van Lommel L. The Vitamin D Analog, TX527, Promotes a Human CD4+CD25highCD127low Regulatory T Cell Profile and Induces a Migratory Signature Specific for Homing to Sites of Inflammation. J Immunol (2011) 186(1):132–42. doi: 10.4049/jimmunol.1000695 21131424

[14] Martens PJ Gysemans C Verstuyf A Mathieu AC. Vitamin D's Effect on Immune Function. Nutrients (2020) 12(5):1248. doi: 10.3390/nu12051248 PMC7281985 32353972

[15] Vanherwegen AS Gysemans C Mathieu C. Regulation of Immune Function by Vitamin D and Its Use in Diseases of Immunity. Endocrinol Metab Clin North Am (2017) 46(4):1061–94. doi: 10.1016/j.ecl.2017.07.010 29080635

[16] Mathieu C Waer M Laureys J Rutgeerts O Bouillon R. Prevention of Autoimmune Diabetes in NOD Mice by 1,25 Dihydroxyvitamin D3. Diabetologia (1994) 37(6):552–8. doi: 10.1007/BF00403372 7926338

[17] Takiishi T Ding L Baeke F Spagnuolo I Sebastiani G Laureys J. Dietary Supplementation With High Doses of Regular Vitamin D3 Safely Reduces Diabetes Incidence in NOD Mice When Given Early and Long Term. Diabetes (2014) 63(6):2026–36. doi: 10.2337/db13-1559 24550187

[18] Hypponen E Laara E Reunanen A Jarvelin MR Virtanen SM. Intake of Vitamin D and Risk of Type 1 Diabetes: A Birth-Cohort Study. Lancet (2001) 358(9292):1500–3. doi: 10.1016/S0140-6736(01)06580-1 11705562

[19] Wicklow BA Taback SP. Feasibility of a Type 1 Diabetes Primary Prevention Trial Using 2000 IU Vitamin D3 in Infants From the General Population With Increased HLA-Associated Risk. Ann N Y Acad Sci (2006) 1079:310–2. doi: 10.1196/annals.1375.047 17130571

[20] Holick MF Binkley NC Bischoff-Ferrari HA Gordon CM Hanley DA Heaney RP. Evaluation, Treatment, and Prevention of Vitamin D Deficiency: An Endocrine Society Clinical Practice Guideline. J Clin Endocrinol Metab (2011) 96(7):1911–30. doi: 10.1210/jc.2011-0385 21646368

[21] Akimbekov NS Digel I Sherelkhan DK Lutfor AB Razzaque MS. Vitamin D and the Host-Gut Microbiome: A Brief Overview. Acta Histochem Cytochem (2020) 53(3):33–42. doi: 10.1267/ahc.20011 32624628 PMC7322162

[22] Singh P Rawat A Alwakeel M Sharif E Al Khodor S. The Potential Role of Vitamin D Supplementation as a Gut Microbiota Modifier in Healthy Individuals. Sci Rep (2020) 10(1):21641. doi: 10.1038/s41598-020-77806-4 33303854 PMC7729960

[23] Ryz NR Lochner A Bhullar K Ma C Huang T Bhinder G. Dietary Vitamin D3 Deficiency Alters Intestinal Mucosal Defense and Increases Susceptibility to Citrobacter Rodentium-Induced Colitis. Am J Physiol Gastrointest Liver Physiol (2015) 309(9):G730–42. doi: 10.1152/ajpgi.00006.2015 PMC4628967 26336925

[24] Yamamoto EA Jorgensen TN. Relationships Between Vitamin D, Gut Microbiome, and Systemic Autoimmunity. Front Immunol (2019) 10:3141. doi: 10.3389/fimmu.2019.03141 32038645 PMC6985452

[25] Bosman ES Albert AY Lui H Dutz JP Vallance BA. Skin Exposure to Narrow Band Ultraviolet (UVB) Light Modulates the Human Intestinal Microbiome. Front Microbiol (2019) 10:2410. doi: 10.3389/fmicb.2019.02410 31708890 PMC6821880

[26] Tangestani H Boroujeni HK Djafarian K Emamat H Shab-Bidar S. Vitamin D and The Gut Microbiota: A Narrative Literature Review. Clin Nutr Res (2021) 10(3):181–91. doi: 10.7762/cnr.2021.10.3.181 PMC8331286 34386438

[27] Wang J Thingholm LB Skieceviciene J Rausch P Kummen M Hov JR. Genome-Wide Association Analysis Identifies Variation in Vitamin D Receptor and Other Host Factors Influencing the Gut Microbiota. Nat Genet (2016) 48(11):1396–406. doi: 10.1038/ng.3695 PMC5626933 27723756

[28] Makishima M Lu TT Xie W Whitfield GK Domoto H Evans RM. Vitamin D Receptor as an Intestinal Bile Acid Sensor. Science (2002) 296(5571):1313–6. doi: 10.1126/science.1070477 12016314

[29] Chatterjee I Lu R Zhang Y Zhang J Dai Y Xia Y. Vitamin D Receptor Promotes Healthy Microbial Metabolites and Microbiome. Sci Rep (2020) 10(1):7340. doi: 10.1038/s41598-020-64226-7 32355205 PMC7192915

[30] National Research Council (US) Subcommittee on Laboratory Animal Nutrition Nutrient Requirements of Laboratory Animals: Fourth Revised Edition, 1995. Washington (DC): National Academies Press (US) (1995). 25121259

[31] Casetta B Jans I Billen J Vanderschueren D Bouillon R. Development of a Method for the Quantification of 1alpha,25(OH)2-Vitamin D3 in Serum by Liquid Chromatography Tandem Mass Spectrometry Without Derivatization. Eur J Mass Spectrom (Chichester) (2010) 16(1):81–9. doi: 10.1255/ejms.1024 20065517

[32] Tito RY Cypers H Joossens M Varkas G Van Praet L Glorieus E. Brief Report: Dialister as a Microbial Marker of Disease Activity in Spondyloarthritis. Arthritis Rheumatol (2017) 69(1):114–21. doi: 10.1002/art.39802 27390077

[33] Hildebrand F Tadeo R Voigt AY Bork P Raes J. LotuS: An Efficient and User-Friendly OTU Processing Pipeline. Microbiome (2014) 2(1):30. doi: 10.1186/2049-2618-2-30 27367037 PMC4179863

[34] Callahan BJ McMurdie PJ Rosen MJ Han AW Johnson AJ Holmes SP. DADA2: High-Resolution Sample Inference From Illumina Amplicon Data. Nat Methods (2016) 13(7):581–3. doi: 10.1038/nmeth.3869 PMC4927377 27214047

[35] Quast C Pruesse E Yilmaz P Gerken J Schweer T Yarza P. The SILVA Ribosomal RNA Gene Database Project: Improved Data Processing and Web-Based Tools. Nucleic Acids Res (2013) 41(Database issue):D590–6. doi: 10.1093/nar/gks1219 PMC3531112 23193283

[36] Love MI Huber W Anders S. Moderated Estimation of Fold Change and Dispersion for RNA-Seq Data With Deseq2. Genome Biol (2014) 15(12):550. doi: 10.1186/s13059-014-0550-8 25516281 PMC4302049

[37] Segata N Izard J Waldron L Gevers D Miropolsky L Garrett WS. Metagenomic Biomarker Discovery and Explanation. Genome Biol (2011) 12(6):R60. doi: 10.1186/gb-2011-12-6-r60 21702898 PMC3218848

[38] McMurdie PJ Holmes S. Phyloseq: An R Package for Reproducible Interactive Analysis and Graphics of Microbiome Census Data. PloS One (2013) 8(4):e61217. doi: 10.1371/journal.pone.0061217 23630581 PMC3632530

[39] Ogle DH DJ Wheeler P Dinno A. FSA: Fisheries Stock Analysis. R. Package Version 0.9.2. Ogle DH. Northland College, Ashland, Wisconsin, US (2022).

[40] RCoreTeam. R: A Language and Environment for Statistical Computing. Vienna, Austria: R Foundation for Statistical Computing (2021).

[41] Wickham H. Ggplot2. WIREs Comput Statistics (2011) 3(2):180–5. doi: 10.1002/wics.147

[42] Deaglio S Dwyer KM Gao W Friedman D Usheva A Erat A. Adenosine Generation Catalyzed by CD39 and CD73 Expressed on Regulatory T Cells Mediates Immune Suppression. J Exp Med (2007) 204(6):1257–65. doi: 10.1084/jem.20062512 PMC2118603 17502665

[43] Gagliani N Magnani CF Huber S Gianolini ME Pala M Licona-Limon P. Coexpression of CD49b and LAG-3 Identifies Human and Mouse T Regulatory Type 1 Cells. Nat Med (2013) 19(6):739–46. doi: 10.1038/nm.3179 23624599

[44] Monsted MO Falck ND Pedersen K Buschard K Holm LJ Haupt-Jorgensen M. Intestinal Permeability in Type 1 Diabetes: An Updated Comprehensive Overview. J Autoimmun (2021) 122:102674. doi: 10.1016/j.jaut.2021.102674 34182210

[45] Kong J Zhang Z Musch MW Ning G Sun J Hart J. Novel Role of the Vitamin D Receptor in Maintaining the Integrity of the Intestinal Mucosal Barrier. Am J Physiol Gastrointest Liver Physiol (2008) 294(1):G208–16. doi: 10.1152/ajpgi.00398.2007 17962355

[46] Bouillon R Marcocci C Carmeliet G Bikle D White JH Dawson-Hughes B. Skeletal and Extraskeletal Actions of Vitamin D: Current Evidence and Outstanding Questions. Endocr Rev (2019) 40(4):1109–51. doi: 10.1210/er.2018-00126 PMC6626501 30321335

[47] Bouillon R Van Schoor NM Gielen E Boonen S Mathieu C Vanderschueren D. Optimal Vitamin D Status: A Critical Analysis on the Basis of Evidence-Based Medicine. J Clin Endocrinol Metab (2013) 98(8):E1283-304. doi: 10.1210/jc.2013-1195 23922354

[48] Ross AC Manson JE Abrams SA Aloia JF Brannon PM Clinton SK. The 2011 Report on Dietary Reference Intakes for Calcium and Vitamin D From the Institute of Medicine: What Clinicians Need to Know. J Clin Endocrinol Metab (2011) 96(1):53–8. doi: 10.1210/jc.2010-2704 PMC3046611 21118827

[49] Heaney RP. Toward a Physiological Referent for the Vitamin D Requirement. J Endocrinol Invest (2014) 37(11):1127–30. doi: 10.1007/s40618-014-0190-6 25308199

[50] Vieth R. Vitamin D Supplementation, 25-Hydroxyvitamin D Concentrations, and Safety. Am J Clin Nutr (1999) 69(5):842–56. doi: 10.1093/ajcn/69.5.842 10232622

[51] Kimball SM Holick MF. Official Recommendations for Vitamin D Through the Life Stages in Developed Countries. Eur J Clin Nutr (2020) 74(11):1514–8. doi: 10.1038/s41430-020-00706-3 32820241

[52] Herrmann M Farrell CL Pusceddu I Fabregat-Cabello N Cavalier E. Assessment of Vitamin D Status - a Changing Landscape. Clin Chem Lab Med (2017) 55(1):3–26. doi: 10.1515/cclm-2016-0264 27362963

[53] Wagner D Hanwell HE Schnabl K Yazdanpanah M Kimball S Fu L. The Ratio of Serum 24,25-Dihydroxyvitamin D(3) to 25-Hydroxyvitamin D(3) is Predictive of 25-Hydroxyvitamin D(3) Response to Vitamin D(3) Supplementation. J Steroid Biochem Mol Biol (2011) 126(3-5):72–7. doi: 10.1016/j.jsbmb.2011.05.003 21605672

[54] Overbergh L Decallonne B Valckx D Verstuyf A Depovere J Laureys J. Identification and Immune Regulation of 25-Hydroxyvitamin D-1-Alpha-Hydroxylase in Murine Macrophages. Clin Exp Immunol (2000) 120(1):139–46. doi: 10.1046/j.1365-2249.2000.01204.x PMC1905630 10759775

[55] Prietl B Treiber G Mader JK Hoeller E Wolf M Pilz S. High-Dose Cholecalciferol Supplementation Significantly Increases Peripheral CD4(+) Tregs in Healthy Adults Without Negatively Affecting the Frequency of Other Immune Cells. Eur J Nutr (2014) 53(3):751–9. doi: 10.1007/s00394-013-0579-6 23999998

[56] Treiber G Prietl B Frohlich-Reiterer E Lechner E Ribitsch A Fritsch M. Cholecalciferol Supplementation Improves Suppressive Capacity of Regulatory T-Cells in Young Patients With New-Onset Type 1 Diabetes Mellitus - A Randomized Clinical Trial. Clin Immunol (2015) 161(2):217–24. doi: 10.1016/j.clim.2015.08.002 26277548

[57] Vignali DA Collison LW Workman CJ. How Regulatory T Cells Work. Nat Rev Immunol (2008) 8(7):523–32. doi: 10.1038/nri2343 PMC2665249 18566595

[58] Jeffery LE Burke F Mura M Zheng Y Qureshi OS Hewison M. 1,25-Dihydroxyvitamin D3 and IL-2 Combine to Inhibit T Cell Production of Inflammatory Cytokines and Promote Development of Regulatory T Cells Expressing CTLA-4 and Foxp3. J Immunol (2009) 183(9):5458–67. doi: 10.4049/jimmunol.0803217 PMC2810518 19843932

[59] Kang SW Kim SH Lee N Lee WW Hwang KA Shin MS. 1,25-Dihyroxyvitamin D3 Promotes FOXP3 Expression *via* Binding to Vitamin D Response Elements in its Conserved Noncoding Sequence Region. J Immunol (2012) 188(11):5276–82. doi: 10.4049/jimmunol.1101211 PMC3358577 22529297

[60] Chauss D Freiwald T McGregor R Yan B Wang L Nova-Lamperti E. Autocrine Vitamin D Signaling Switches Off Pro-Inflammatory Programs of TH1 Cells. Nat Immunol (2022) 23(1):62–74. doi: 10.1038/s41590-021-01080-3 34764490 PMC7612139

[61] Nanzer AM Chambers ES Ryanna K Richards DF Black C Timms PM. Enhanced Production of IL-17A in Patients With Severe Asthma is Inhibited by 1alpha,25-Dihydroxyvitamin D3 in a Glucocorticoid-Independent Fashion. J Allergy Clin Immunol (2013) 132(2):297–304 e3. doi: 10.1016/j.jaci.2013.03.037 23683514

[62] Mann EH Chambers ES Chen YH Richards DF Hawrylowicz CM. 1alpha,25-Dihydroxyvitamin D3 Acts *via* Transforming Growth Factor-Beta to Up-Regulate Expression of Immunosuppressive CD73 on Human CD4+ Foxp3- T Cells. Immunology (2015) 146(3):423–31. doi: 10.1111/imm.12519 PMC4610631 26251265

[63] Thiel M Caldwell CC Sitkovsky MV. The Critical Role of Adenosine A2A Receptors in Downregulation of Inflammation and Immunity in the Pathogenesis of Infectious Diseases. Microbes Infect (2003) 5(6):515–26. doi: 10.1016/S1286-4579(03)00068-6 12758281

[64] Tai N Wong FS Wen L. TLR9 Deficiency Promotes CD73 Expression in T Cells and Diabetes Protection in Nonobese Diabetic Mice. J Immunol (2013) 191(6):2926–37. doi: 10.4049/jimmunol.1300547 PMC3788667 23956420

[65] Sorini C Cosorich I Lo Conte M De Giorgi L Facciotti F Luciano R. Loss of Gut Barrier Integrity Triggers Activation of Islet-Reactive T Cells and Autoimmune Diabetes. Proc Natl Acad Sci U S A (2019) 116(30):15140–9. doi: 10.1073/pnas.1814558116 PMC6660755 31182588

[66] Tan J McKenzie C Potamitis M Thorburn AN Mackay CR Macia L. The Role of Short-Chain Fatty Acids in Health and Disease. Adv Immunol (2014) 121:91–119. doi: 10.1016/B978-0-12-800100-4.00003-9 24388214

[67] Fiorucci S Biagioli M Zampella A Distrutti E. Bile Acids Activated Receptors Regulate Innate Immunity. Front Immunol (2018) 9:1853. doi: 10.3389/fimmu.2018.01853 30150987 PMC6099188

[68] Ding RX Goh WR Wu RN Yue XQ Luo X Khine WWT. Revisit Gut Microbiota and its Impact on Human Health and Disease. J Food Drug Anal (2019) 27(3):623–31. doi: 10.1016/j.jfda.2018.12.012 PMC9307029 31324279

[69] Giongo A Gano KA Crabb DB Mukherjee N Novelo LL Casella G. Toward Defining the Autoimmune Microbiome for Type 1 Diabetes. ISME J (2011) 5(1):82–91. doi: 10.1038/ismej.2010.92 20613793 PMC3105672

[70] Stene LC Magnus P Lie RT Sovik O Joner G. Norwegian Childhood Diabetes Study G. No Association Between Preeclampsia or Cesarean Section and Incidence of Type 1 Diabetes Among Children: A Large, Population-Based Cohort Study. Pediatr Res (2003) 54(4):487–90. doi: 10.1203/01.PDR.0000081301.25600.5D 12815116

[71] Kahalehili HM Newman NK Pennington JM Kolluri SK Kerkvliet NI Shulzhenko N. Dietary Indole-3-Carbinol Activates AhR in the Gut, Alters Th17-Microbe Interactions, and Exacerbates Insulitis in NOD Mice. Front Immunol (2020) 11:606441. doi: 10.3389/fimmu.2020.606441 33552063 PMC7858653

[72] Jacob N Jaiswal S Maheshwari D Nallabelli N Khatri N Bhatia A. Butyrate Induced Tregs are Capable of Migration From the GALT to the Pancreas to Restore Immunological Tolerance During Type-1 Diabetes. Sci Rep (2020) 10(1):19120. doi: 10.1038/s41598-020-76109-y 33154424 PMC7644709

[73] Bashir M Prietl B Tauschmann M Mautner SI Kump PK Treiber G. Effects of High Doses of Vitamin D3 on Mucosa-Associated Gut Microbiome Vary Between Regions of the Human Gastrointestinal Tract. Eur J Nutr (2016) 55(4):1479–89. doi: 10.1007/s00394-015-0966-2 PMC4875045 26130323

