# Supplementary material for: High Serum Vitamin D Concentrations, Induced via Diet, Trigger Immune and Intestinal Microbiota Alterations Leading to Type 1 Diabetes Protection in NOD Mice
Source: Front Immunol. 2022 Jun 9;13:902678. doi: 10.3389/fimmu.2022.902678 (PMC9241442; doi:10.3389/fimmu.2022.902678)
Supplement: Supplementary file 1 [file DataSheet_1.pdf]

Electronic Supplementary Material

Supplementary Figure 1

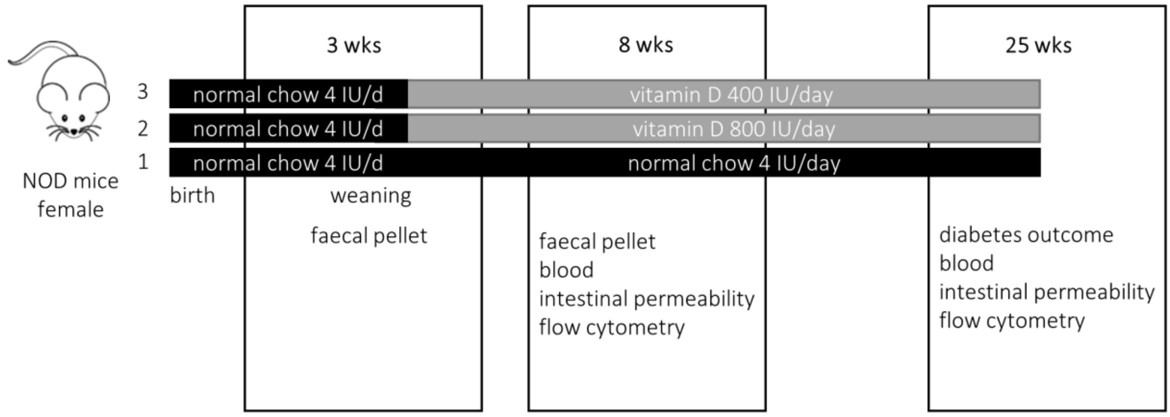

Supplementary Figure 1. Vitamin D substitution regimen in prevention of T1D in NOD mice. Female NOD mice, 3 weeks of age, were randomly assigned to 3 dietary groups until 25 weeks of age: (**1**) normal chow (NC) containing 1% calcium, 0,7% phosphorus and 1,000 IU vitamin D/kg diet; (**2**) 400 IU/day vitamin D-supplemented diet containing 1% calcium, 0,7% phosphorus and 100,000 IU vitamin D_3/kg diet; and (**3**) 800 IU/day vitamin D-supplemented containing 1% calcium, 0,7% phosphorus and 200,000 IU vitamin D/kg diet. At 3 and 8 weeks of age, faecal pellets were collected for 16sRNA sequencing. At 8 and 25 weeks of age, blood sampling, gut epithelial barrier function, and flow cytometry were scheduled. All mice were monitored 3 times weekly for diabetes development until 25 weeks of age.

Supplementary Figure 2

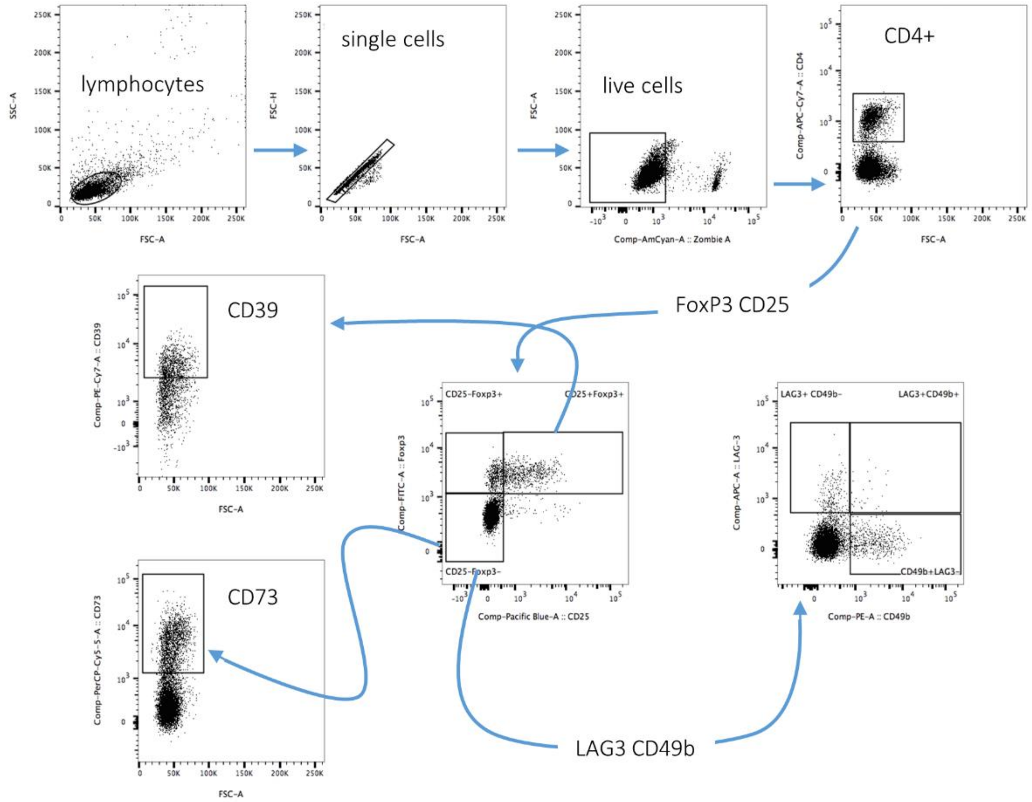

Supplementary Figure 2. Gating strategy to identify Treg and Tr1 population in spleen, PLN, MLN in pre-diabetic NOD mice. Single-cell suspensions from NOD mice (both 8 and 25 weeks of age) were stained with a combination of 7 antibodies and a viable dye as described in the experimental design and methods section. Lymphocytes were identified based on their forward- and side-scatter properties. Subsequently, dead cells were excluded through the use of a viability dye. Treg cells were identified as FoxP3$^+$CD4$^+$ T cells, having either CD25$^+$ or CD25$^-$ expression levels. Tr1 cells, which have been shown to express high levels of the immunosuppressive cytokine IL-10 but not transcription factor FoxP3, are identified as LAG3$^+$CD49b$^+$ within the FoxP3$^-$CD4$^+$ T-cell gate. The cell surface ecto-enzymes CD39 and CD73 regulate levels of ATP and adenosine, and therefore inflammation, by dephosphorylating ATP into ADP and AMP, and then into adenosine. Treg cells have been shown to express CD73 as a novel marker. Treg cells expressing CD39 or CD73 were identified as CD39$^+$, CD73$^+$, or CD39$^+$CD73$^+$ within the FoxP3$^+$CD4$^+$ T-cell gate, respectively.

Supplementary Figure 3

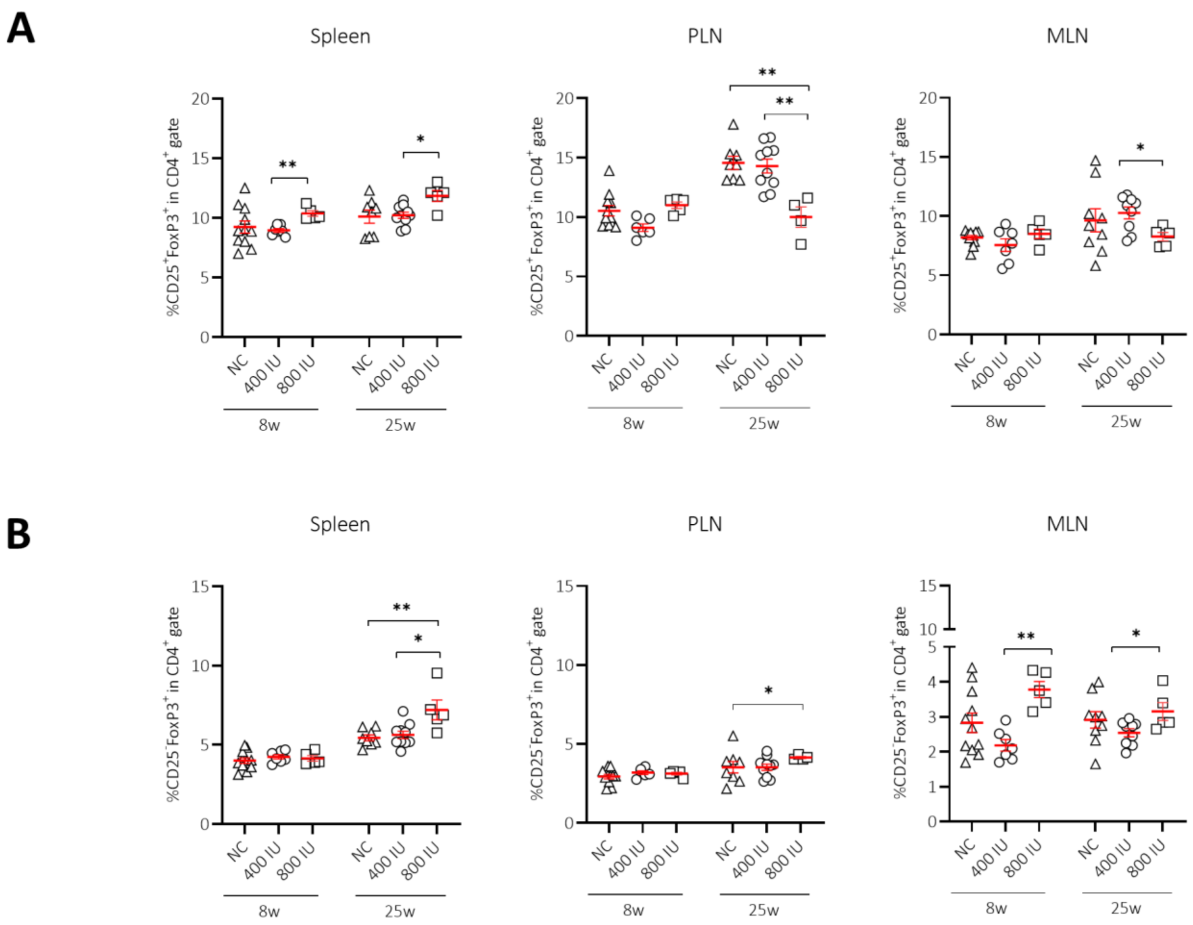

Supplementary Figure 3. Effect of vitamin D substitution regimen on frequency of $CD4^+CD25^+FoxP3^+$ and $CD4^+CD25^-FoxP3^+$ Treg cells. Frequency of $CD25^+FoxP3^+$ (**A**) or $CD25^-FoxP3^+$ (**B**) cells within $CD4^+$ T-cell gate are shown at both 8 and 25 weeks of age in spleen, pancreatic draining lymph nodes (PLN), mesenteric lymph nodes (MLN). Female NOD mice were fed normal chow (NC) or two different doses of vitamin D-supplemented (400 IU/day or 800 IU/day) diet from 3 until 25 weeks of age (lifelong). Symbols (N = 4-11) represent individual mice, and line reflects group mean with SEM. *P<0.05; **P≤0.01.

Supplementary Table S1

								Chao1	Shannon
Diet	Test	Age	Outcome	Group1	Group2	N group1	N group2	P value	
NC	paired MWU	3 vs 8w	P	3w	8w	9	9	0,138	0,359
400 IU	paired MWU	3 vs 8w	P	3w	8w	5	5	0,100	0,188
800 IU	paired MWU	3 vs 8w	P	3w	8w	4	4	0,181	0,625
NC	paired MWU	3 vs 8w	NP	3w	8w	10	10	0,066	0,006
400 IU	paired MWU	3 vs 8w	NP	3w	8w	5	5	0,313	0,063
800 IU	paired MWU	3 vs 8w	NP	3w	8w	5	5	0,100	0,625
NC	MWU	8w	P vs NP	NP	P	10	9	0,008	0,182
400 IU	MWU	8w	P vs NP	NP	P	5	5	0,033	0,222
800 IU	MWU	8w	P vs NP	NP	P	5	4	0,049	0,286
NC, 400 IU, 800 IU	KWT	8w	P and NP	NC, 400 IU, 800 IU		38		0,794	0,291

Supplementary Table S1. Changes in alpha-diversity indices between 3 and 8 weeks of age in NOD mice progressing or not progressing towards T1D (paired Mann-Whitney U test). Comparison of Shannon and Chao1 indices at 8 weeks of age between T1D progressor (P) and non-progressor (NP) NOD mice receiving normal chow (NC), the 400 or 800 IU/day vitamin D-supplemented diets (Mann–Whitney U-test; MWU). Comparison of Shannon and Chao1 indices at 8 weeks of age between NOD mice receiving NC, the 400 or 800 IU/day vitamin D-supplemented diets (Kruskal–Wallis test; KWT). Statistically significant features ($P \leq 0.05$) are indicated in red.

Supplementary Table S2

A.

Diet	Test	Age	Outcome	Group1	Group2	N group1	N group2	*Actinobacteria*	*Bacteroidetes*	*Epsilonbacteraeota*	*Firmicutes*	*Proteobacteria*	*Tenericutes*	*Verrucomicrobia*	*Bacteroidetes/Firmicutes*
								P value							
NC	paired MWU	3 vs 8w	P	3w	8w	9	9	0,129	0,141	0,074	0,910	0,036	0,098	0,933	1,000
400 IU	paired MWU	3 vs 8w	P	3w	8w	5	5	0,125	0,789	1,000	1,000	0,063	0,313	1,000	1,000
800 IU	paired MWU	3 vs 8w	P	3w	8w	4	4	0,625	0,423	1,000	0,625	0,125	0,789	0,250	0,625
NC	paired MWU	3 vs 8w	NP	3w	8w	10	10	0,105	0,375	0,018	0,553	0,064	0,049	0,673	0,922
400 IU	paired MWU	3 vs 8w	NP	3w	8w	5	5	0,188	0,855	0,813	0,063	0,125	0,125	0,100	0,125
800 IU	paired MWU	3 vs 8w	NP	3w	8w	5	5	0,125	0,063	0,063	0,855	0,063	0,063	0,625	0,313
NC	MWU	8w	P vs NP	NP	P	10	9	0,182	0,346	1,000	0,089	0,111	0,497	0,932	0,264
400 IU	MWU	8w	P vs NP	NP	P	5	5	0,690	0,824	0,841	0,173	0,548	0,548	0,824	0,481
800 IU	MWU	8w	P vs NP	NP	P	5	4	0,190	0,533	0,111	0,306	0,190	0,032	0,227	0,408
NC, 400 IU, 800 IU	KWT	8w	P and NP	NC, 400 IU, 800 IU		38		0,297	0,003	0,015	0,525	0,106	0,740	0,589	0,038

B.

Diet	Test	Age	Group1	Group2	N group1	N group2	P value *Bacteroidetes*	P value *Epsilonbacteraeota*	P value *Bacteroidetes/Firmicutes*
NC vs 400 IU	post-hoc Dunn's test	8w	NC	400 IU	19	10	0,008	0,006	0,105
NC vs 800 IU	post-hoc Dunn's test	8w	NC	800 IU	19	10	0,317	0,089	0,186
400 vs 800 IU	post-hoc Dunn's test	8w	400 IU	800 IU	10	9	0,002	0,390	0,011

Supplementary Table S2. Changes in phyla abundances between 3 and 8 weeks of age in faecal samples of mice progressing or not progressing towards T1D (paired Mann-Whitney U test). Comparison of genera abundances at 8 weeks of age between T1D progressor (P)and non-progressor (NP) NOD mice receiving NC, the 400 or 800 IU/day vitamin D-supplemented diets (Mann–Whitney U-test; MWU)(**A**). Comparison of genera abundances at 8 weeks of age between NOD mice receiving NC, the 400 or 800 IU/day vitamin D-supplemented diets (Kruskal–Wallis; KWT with post-hoc Dunn's test)(**B**). Statistically significant features ($P \leq 0.05$) are indicated in red.

Supplementary Table S3

A

								g_A2	*g_Acetatifactor*	*g_Akkermansia*	*g_Anaerotruncus*	*g_Bacteroides*	*g_Butyricicoccus*	*g_Enterorhabdus*	*g_Erysipelatoclostridium*	*g_GCA-900066575*	*g_Helicobacter*	*g_Hydrogenoanaerobacterium*	*g_Intestinimonas*
Diet	Test	Age	Outcome	group1	group2	N group1	N group2	P value											
NC	paired MWU	3 vs 8w	P	3w	8w	9	9	0,652	0,496	0,554	0,129	0,820	0,080	0,129	1,000	0,944	0,055	0,652	0,250
NC	paired MWU	3 vs 8w	NP	3w	8w	10	10	0,492	0,131	0,800	0,625	0,432	0,554	0,922	0,770	0,770	0,014	0,084	0,846
NC	MWU	8w	P vs NP	NP	P	10	9	0,462	0,780	0,865	0,497	0,315	0,712	0,549	0,079	0,780	0,780	0,780	0,447
400 IU	MWU	8w	P vs NP	NP	P	5	5	0,675	0,310	0,824	0,421	0,690	0,600	0,548	0,917	0,056	0,690	0,841	0,421
800 IU	MWU	8w	P vs NP	NP	P	5	4	0,111	0,413	0,227	0,111	1,000	0,063	0,556	0,712	0,286	0,730	0,111	0,413
NC, 400, and 800 IU	KWT	8w	P and NP	NC, 400 IU, 800 IU		38		0,707	0,790	0,589	0,362	0,188	0,110	0,303	0,433	0,149	0,102	0,886	0,561

B

Diet	Test	Age	Outcome	Group1	Group2	N group1	N group2	*g_Lachnoclostridium*	*g_Lachnospiraceae_FCS020_group*	*g_Lachnospiraceae_NK4A136_group*	*g_Lachnospiraceae_UCG-001*	*g_Lachnospiraceae_UCG-006*	*g_Lactobacillus*	*g_Marvinbryantia*	*g_Muribaculum*	*g_Oscillibacter*	*g_Roseburia*	*g_Ruminiclostridium*	*g_Ruminiclostridium_5*
								P value											
NC	paired MWU	3 vs 8w	P	3w	8w	9	9	0,426	0,820	0,570	0,441	0,203	0,910	0,820	0,301	0,301	0,496	0,910	0,359
NC	paired MWU	3 vs 8w	NP	3w	8w	10	10	0,375	0,432	0,625	0,044	1,000	0,193	0,407	0,275	0,695	0,922	0,695	0,160
NC	MWU	8w	P vs NP	NP	P	10	9	0,133	0,513	0,720	0,806	0,842	0,497	0,744	0,968	0,156	0,661	0,842	0,079
400 IU	MWU	8w	P vs NP	NP	P	5	5	1,000	0,841	0,690	0,056	0,421	0,690	0,841	0,690	0,421	0,421	0,222	0,151
800 IU	MWU	8w	P vs NP	NP	P	5	4	1,000	0,806	0,063	0,171	0,063	1,000	0,502	0,730	0,730	0,730	0,905	0,190
NC, 400, and 800 IU	KWT	8w	P and NP	NC, 400 IU, 800 IU		38		0,861	0,009	0,199	0,883	0,264	0,066	≤ 0,001	0,258	0,887	0,114	0,070	0,306

C.

								g_Ruminiclostridium_9	*g_Ruminococcaceae_UCG-014*	*uc_f_Clostridiales_vadinBB60_group*	*uc_f_Eggerthellaceae*	*uc_f_Erysipelotrichaceae*	*uc_f_Family_XIII*	*uc_f_Lachnospiraceae*	*uc_f_Muribaculaceae*	*uc_f_Peptococcaceae*	*uc_f_Ruminococcaceae*	*uc_o_Mollicutes_RF39*
Diet	Test	Age	Outcome	Group1	Group2	N group1	N group2	P value										
NC	paired MWU	3 vs 8w	P	3w	8w	9	9	0,570	1,000	0,496	0,820	0,301	0,734	0,250	0,734	1,000	0,570	0,359
NC	paired MWU	3 vs 8w	NP	3w	8w	10	10	0,695	0,432	0,625	0,275	0,770	0,064	0,922	0,695	0,322	0,193	0,084
NC	MWU	8w	P vs NP	NP	P	10	9	0,400	0,604	0,053	0,111	0,079	0,236	0,400	0,842	0,153	0,720	0,315
400 IU	MWU	8w	P vs NP	NP	P	5	5	0,421	0,310	1,000	1,000	0,841	0,421	0,690	1,000	0,095	0,548	0,690
800 IU	MWU	8w	P vs NP	NP	P	5	4	0,063	0,556	0,730	0,065	1,000	0,905	0,905	0,905	0,556	0,111	0,110
NC, 400, and 800 IU	KWT	8w	P and NP	NC, 400 IU, 800 IU		38		0,019	0,925	0,215	0,398	0,578	0,184	0,063	0,142	0,242	0,171	0,168

D

Diet	Test	Age	Group1	Group2	N group1	N group2	P value *g_Lachnospiraceae_FCS020_g*	*g_Marvinbryantia*	*g_Ruminiclostridium_9*
NC vs 400 IU	post-hoc Dunn's test	8w	NC	400 IU	19	10	0,003	0,134	0,728
NC vs 800 IU	post-hoc Dunn's test	8w	NC	800 IU	19	10	0,714	≤ 0,001	0,012
400 vs 800 IU	post-hoc Dunn's test	8w	400 IU	800 IU	10	9	0,026	0,015	0,012

Supplementary Table S3. Changes in genera abundances between 3 and 8 weeks of age in faecal samples of mice progressing or not progressing towards T1D. Comparison of genera abundances at 8 weeks of age between T1D progressor (P) and non-progressor (NP) NOD mice receiving NC, the 400 or 800 IU/day vitamin D-supplemented diets (Mann–Whitney U test; MWU)(**A**-**C**). Comparison of genera abundances at 8 weeks of age between NOD mice receiving NC, the 400 or 800 IU/day vitamin D-supplemented diets (Kruskal–Wallis; KWT with post-hoc Dunn's test)(**D**). Statistically significant features (P ≤ 0.05) are indicated in red.
